# Control of sustained attention and impulsivity by G_q_-protein signalling in parvalbumin interneurons of the anterior cingulate cortex

**DOI:** 10.1038/s41398-023-02541-z

**Published:** 2023-07-05

**Authors:** Martin M. Jendryka, Uwe Lewin, Bastiaan van der Veen, Sampath K. T. Kapanaiah, Vivien Prex, Daniel Strahnen, Thomas Akam, Birgit Liss, Anton Pekcec, Wiebke Nissen, Dennis Kätzel

**Affiliations:** 1 Institute of Applied Physiology, Ulm University, Ulm, Germany; 2 Boehringer Ingelheim Pharma GmbH & Co. KG, Div. Research Germany, Biberach an der Riss, Germany; 3 Department of Experimental Psychology and Wellcome Centre for Integrative Neuroimaging, University of Oxford, Oxford, UK; 4 Linacre College and New College, University of Oxford, Oxford, UK

**Keywords:** Molecular neuroscience, ADHD

## Abstract

The anterior cingulate cortex (ACC) has been implicated in attention deficit hyperactivity disorder (ADHD). More specifically, an appropriate balance of excitatory and inhibitory activity in the ACC may be critical for the control of impulsivity, hyperactivity, and sustained attention which are centrally affected in ADHD. Hence, pharmacological augmentation of parvalbumin- (PV) or somatostatin-positive (Sst) inhibitory ACC interneurons could be a potential treatment strategy. We, therefore, tested whether stimulation of G_q_-protein-coupled receptors (G_q_PCRs) in these interneurons could improve attention or impulsivity assessed with the 5-choice-serial reaction-time task in male mice. When challenging impulse control behaviourally or pharmacologically, activation of the chemogenetic G_q_PCR hM3Dq in ACC PV-cells caused a selective decrease of active erroneous—i.e. incorrect and premature—responses, indicating improved attentional and impulse control. When challenging attention, in contrast, omissions were increased, albeit without extension of reward latencies or decreases of attentional accuracy. These effects largely resembled those of the ADHD medication atomoxetine. Additionally, they were mostly independent of each other within individual animals. G_q_PCR activation in ACC PV-cells also reduced hyperactivity. In contrast, if hM3Dq was activated in Sst-interneurons, no improvement of impulse control was observed, and a reduction of incorrect responses was only induced at high agonist levels and accompanied by reduced motivational drive. These results suggest that the activation of G_q_PCRs expressed specifically in PV-cells of the ACC may be a viable strategy to improve certain aspects of sustained attention, impulsivity and hyperactivity in ADHD.

## Introduction

Attention-deficit hyperactivity disorder (ADHD) and related impulse control disorders are characterised by high impulsivity, hyperactivity, and difficulty to exert and sustain attentional focus (vigilance) [1–4]. While these symptoms may respond to psychostimulants and partially to noradrenergic medication, such treatment options are rather unspecific and associated with unwanted side effects like sedation or risk of abuse [2, 4, 5], calling for a renewed drug discovery effort. At the neurobiological level, structural or physiological abnormalities in the prefrontal (PFC) and anterior cingulate cortex (ACC), as well as in the parts of the striatum that are innervated by them, have repeatedly been associated with ADHD directly or with its associated deficits in humans and rodent models [3, 6]. Such evidence points particularly to the ACC as being relevant to all three cardinal symptoms of ADHD, i.e. inattention, impulsivity and hyperactivity [6–8]. In humans, transient cessation of psychostimulant medication in ADHD patients led to increased baseline activity in the dorsal ACC [9]. During several impulsivity-challenging tasks, in turn, the dorsal ACC of ADHD patients fails to be activated to a similar degree as seen in control subjects, while the more rostral perigenual ACC is rather hyperactive [6, 10, 11]. This points to a deficit in the appropriate regulation of excitation in the ACC as a potential underpinning of ADHD symptoms.

In line with this evidence, we found recently that the chemogenetic activation of G_i_-protein coupled receptors (G_i_PCRs) in excitatory cells of layer 5 of the mouse ACC can reduce challenge-induced motor impulsivity and novelty-induced hyperactivity [12]. While this evidence points to a potential therapeutic intervention by targeting endogenously expressed G_i_PCRs in layer-5 excitatory cells of the ACC, modulation of GABAergic interneurons that control and temporally shape the activity of ACC excitatory cells might be an alternative therapeutic concept. Importantly, lower GABA levels in the ACC correlate with higher motor impulsivity in adolescent subjects [13]. Further, rats bred for high trait-impulsivity show decreased GABA_A_ receptor binding, specifically in the ACC [14]. These findings suggest that, theoretically, an increase of GABAergic inhibition in the ACC might reduce impulsivity across mammals, but which type of inhibitory interneurons is relevant, which form of their activation is therapeutically effective, and to what extent this intervention would also benefit attentional deficits and hyperactivity, remains elusive.

To answer these questions, we used chemogenetic designer-receptors-exclusively-activated-by-designer-drugs (DREADDs) that can trigger the excitatory G_q_-protein cascade, in order to investigate the behavioural consequences of targeted selective activation of either of the two main types of interneurons that inhibit excitatory pyramidal cells in cortical circuits; parvalbumin-positive (PV) and somatostatin-positive (Sst) interneurons [15, 16]. Aspects of sustained attention and impulse control can be assessed simultaneously by the five-choice serial reaction time task (5-CSRTT) in humans and rodents [17, 18]. This task requires subjects to temporarily withhold poking and detect briefly presented cues to which they need to respond in order to gain rewards. The brevity of stimulus presentation requires continuously high vigilance in order to not miss individual stimuli, and the resulting ratio of correct to the sum of correct and incorrect responses—termed accuracy—is used as a primary measure of sustained attention in the rodent version of the task [17]. Meanwhile, the delay with which the stimulus is presented demands high impulse control to refrain from exploratory, premature poking into any of the holes, and the ratio of such premature responses relative to all trials of a session (%prematures) is used as the primary indicator of motor impulsivity, or impulse control [17, 19]. A previous study found that optogenetic stimulation of PV-cells in the broader dorsal prefrontal region at low-gamma (30–40 Hz) frequency resulted in a decrease of omissions, which represent failures to respond at all (indicating reduced task engagement or inattention), but slower stimulation (1–10 Hz) actually increased omissions and also premature responses [20]. A similar frequency-specific cognition-enhancing effect of prefrontal PV-interneuron stimulation has been found in an assay of cognitive flexibility [21]. Given this limited and frequency-specific efficacy of the activation of PV-cells in such cognitive tasks, it remains unclear whether pharmacological (and, hence, continuous) stimulation of prefrontal PV-interneurons, e.g. through activation of endogenous G_q_-protein-coupled receptors (G_q_PCRs) can actually improve high-level cognition.

## Materials and methods

### Animals and surgery

All experiments were performed in accordance with the German Animal Rights Law (Tierschutzgesetz) 2013 and were approved by the Federal Ethical Review Committee (Regierungsprädsidium Tübingen) of Baden-Württemberg, Germany (licence numbers TV1344 and TV16-017-G). In total, 32 C57BL/6 J male wildtype mice (termed WT cohort), 76 male B6;129P2-Pvalb^tm1(cre)Arbr^/J (PV-Cre, stock# 008069, The Jackson Laboratory, ME, US) [22], including two main behavioural cohorts (termed PV-Gi and PV-Gq, see below), and 38 male STOCK.Sst^tm2.1(cre)Zjh^/J (Sst-Cre, stock# 013044, The Jackson Laboratory) [23] mice, including one behavioural cohort (Sst-Gq), were used for this study. Sample sizes were chosen to be around ten per subgroup after excluding mice with inappropriate viral transfections (see below) based on our prior related chemogenetic study [12].

Mice were pre-trained in the 5-CSRTT (see below) and then assigned to either the control or the DREADD group of their respective cohort, based on their prior attentional performance as a measure of counter-balancing. Stereotactic surgeries were performed as previously described [12] to transduce both the dorsal Cg1 and the ventral Cg2 region of the ACC specifically and bilaterally with an AAV8-vector expressing either mCherry (hSyn-DIO-mCherry; control groups) or a DREADD-mCherry fusion protein (hSyn-DIO-hM3Dq-mCherry or hSyn-DIO-hM4Di-mCherry for Gq- or Gi-DREADD groups, respectively) Cre-dependently [24]. The coordinates were AP + 0.7, ML 0.3, DV 1.65 (200 nl) and 1.3 (300 nl) for the posterior site and AP 1.8, ML 0.25, DV 1.25 (80 nl) for the anterior site; see Supplementary Methods for further surgery details. Mice received post-operative care and were kept on *ad libitum* food for a minimum of two weeks before training in the 5-CSRTT commenced. The WT cohort did not undergo surgery. Animals were group-housed (2–5) in Type II-Long individually ventilated cages (Greenline, Tecniplast, G), enriched either with sawdust, sizzle-nest^TM^, and cardboard houses (Datesand, UK), and maintained at a 13 h light/11 h dark cycle (PV-Gq and Sst-Gq cohorts, see below), or with sawdust, cellulose litter, and polycarbonate houses and maintained at a 12 h light/12 h dark cycle (PV-Gi and WT cohorts). All Cre-transgenic mice were bred from homozygous x C57BL/6 J crosses and were hence heterozygous for Cre.

### Behavioural training and testing

**Fig. 1:**
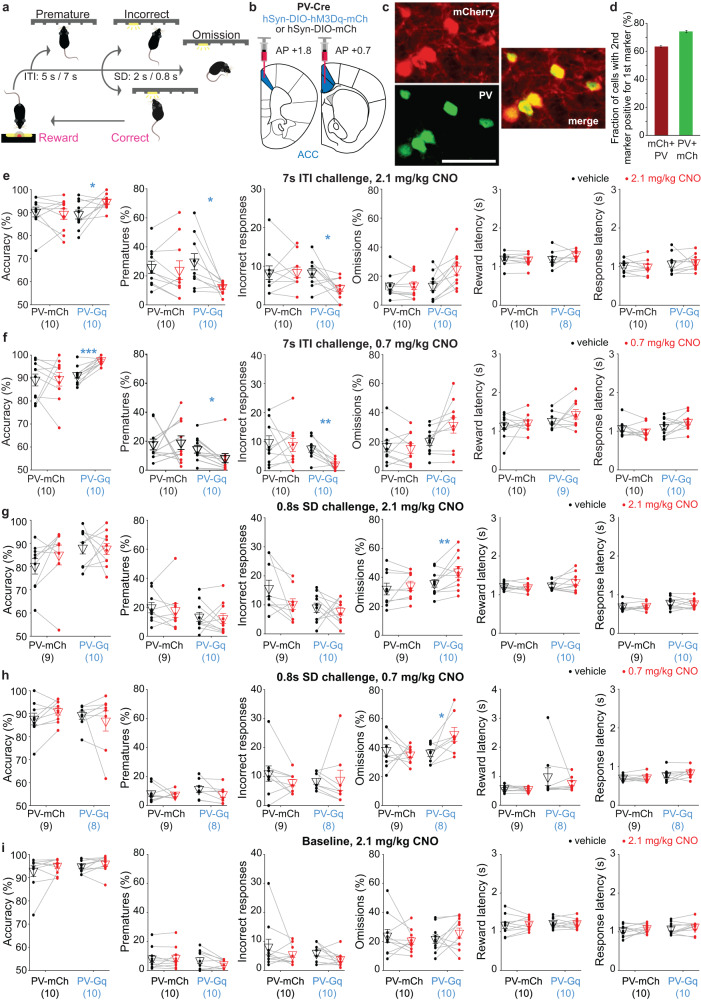
Challenge-specific reduction of premature and incorrect responding by ACC PV-interneuron activation. **a**, **b** Schematic of 5-CSRTT (**a**) and viral transfection (**b**). **c** Example microscopic images showing expression of hM3Dq-mCherry (red) and PV (green). Scale bar, 50 μm. See also Supplementary Fig. 1. **d** Co-expression of mCherry in PV-positive cell (red) and the reverse (green). **e**–**i** Attentional accuracy, premature responding, number of incorrect responses per session, omission rate, and reward and response latency (from left to right, as stated on y-axes) in all behavioural and pharmacological conditions tested in the PV-Gq cohort, as indicated above each row of panels; plotted for each individual mouse for pretreatment with CNO (red dots) or vehicle (black dots) of the groups stated on the x-axes (*N*-numbers in brackets). Blue and black symbols above the data lines indicate significant within-subject differences between vehicle and CNO pretreatment (paired *t*-test). The order, in which the protocols were conducted in this cohort is (**e**–**i**). See also Supplementary Fig. 4 and Supplementary Tables 2, 3 for further data and analysis, including RM-ANOVA. *P < 0.05; **P < 0.01; ***P ≤ 0.001. Non-significant pairwise comparisons (*P* > 0.05) are not indicated. Error bars represent s.e.m. throughout.

Behavioural training and testing with chemogenetic modulation in the 5-CSRTT were done as previously described [12], with some minor modifications in the PV-Gi and WT cohort (see Supplementary Methods). Briefly, mice started training in the 5-CSRTT at 2–3 months of age and were kept under food restriction at 85–95% of their average free-feeding weight measured over 3 days prior to food restriction, with water available ad libitum. Food restriction was interrupted for about 3 weeks around the surgery, and a new baseline weight was used as it was resumed. The training was conducted in 30 min sessions on 5–7 days per week in sound- and light-insulated mouse operant chambers, which were either custom-built featuring classical poke-holes (PV-Gq and Sst-Gq cohorts; described in detail on https://github.com/KaetzelLab/Operant-Box-Design-Files) or, for logistic reasons, in touchscreen-based 5-choice mouse operant chambers (Campden Instruments, Cambridge, UK; PV-Gi and WT cohort). Mice were first trained to poke a hole in the 5-choice wall to obtain a reward (habituation training) and were subsequently trained in the 5-CSRTT over five stages of increasing difficulty (see Supplementary Table 1 for task parameters and criteria for transition between stages). Briefly, a single trial of the 5-CSRTT started with a waiting time (inter-trial-interval, ITI) of either 2 s (stages 1–2) or 5 s (stages 3–5) duration, followed by the illumination of one of the apertures of the 5-choice walls for a certain stimulus duration (SD) of either 20 s (stage 1), 8 s (stages 2-3), 4 s (stage 4) or 2 s (stage 5) indicating to the subject that it has to poke into that aperture (*correct response*) to earn a 20 μl reward (strawberry milk, Müllermilch^TM^, G) which was provided at the reward receptacle on the opposite side of the operant box (Fig. 1a). If mice either poked into any hole during the ITI (*premature response*), poked into a non-illuminated hole (incorrect response) during the SD or limited-hold time (LH, until 2 s after SD), or failed to poke throughout the trial (*omission*), trials were not rewarded but instead terminated immediately with a time-out period during which the house light was turned off. The relative numbers of such response types were used as performance indicators measuring premature responding, i.e. motor impulsivity [%premature = 100*(number of premature responses)/(number of trials)], sustained attention [attentional accuracy = 100*(number of correct responses)/(number of correct and incorrect responses combined)], and *t*ask engagement [%omissions = 100*(number of omissions)/(number of correct, incorrect and omitted responses)]. Also, the time required to poke into the indicated hole after it was illuminated (response latency) and the time from the exit from the correct hole until the entry into the reward receptacle (reward latency) were measured, whereby the latter is usually used as a compound indicator of motivation and locomotor drive [17]. After a minimum of 4 weeks after surgery, a series of multiple 5-CSRTT challenge protocols were conducted in addition to the baseline protocol (equating stage 5) on separate test days (see Supplementary Table 1): either the SD was reduced to 0.8 s to demand high levels of sustained attention (as otherwise the brief stimulus would mostly be missed) or the ITI was extended to 7 or 9 s in order to demand high levels of both impulse control (action postponing) to refrain from premature responding and of sustained attention due to the longer time for which high vigilance was necessary [25, 26].

After testing in the 5-CSRTT was completed, locomotor activity in novel open-fields was measured over 40 min with concomitant chemogenetic modulation as described before [12] and in Supplementary Methods. All experiments were done blind to the subgroup identity of the mice. DREADD-mCherry expression was evaluated histologically post-mortem [12] (see Supplementary Methods) and animals without bilateral expression in the majority of ACC volume or with bilateral expression in the majority of the volume of another brain region were excluded from the dataset.

### Chemogenetic modulation and pharmacology

All compounds applied during behavioural experiments were delivered by either s.c. (Ro63-1908) or i.p. (DREADD agonists, atomoxetine) route at an injection volume of 10 μl/g mouse. For chemogenetic manipulations, the DREADD agonists clozapine-N-oxide (CNO, HB6149, HelloBio, UK, or BML-NS105, Enzo Lifesciences, NY USA), clozapine (CLZ, Sigma-Aldrich, Germany), or vehicle were injected i.p. 10–15 min prior to the start of behavioural testing [27]. For the PV-Gi cohort, both drugs were used as freebase and dissolved in hydrochloric acid (1:15) and 40% hydroxypropyl-β-cyclodextrin (1:10), pH adjusted to 6.5–7.5, as vehicle. In the other cohorts, the dihydrochloride salt of CNO was used and diluted in normal saline as vehicle, whereby the stated doses correspond to the calculated CNO freebase component. Locomotor activity was tested in a between-subjects design (all mice receiving CNO), while all 5-CSRTT experiments were conducted with vehicle and drug as within-subject conditions distributed in a latin-square design counter-balanced within each subgroup across consecutive test days with the same challenge protocol [12]. These consecutive test days were spaced mostly 7 d apart (range: 3–8 d) in all cohorts, and were separated from other experiments in different challenge conditions by at least one week (see Supplementary Table 1 for details). CNO doses were chosen according to prior chemogenetic studies demonstrating effective activation of hM4Di in PV-cells by 10 mg/kg [28–31] and of hM3Dq in PV- and Sst-cells by 1 mg/kg CNO in rodents [28, 32–36], around which we chose our starting doses for hM3Dq (0.7 and 2.1 mg/kg). As a pharmacological model of increased impulsivity, 3 mg/kg of the GluN2B-containing NMDA glutamate receptor antagonist Ro63-1908 [12, 37] was applied s.c. 30 min before behavioural testing. The WT cohort was injected with atomoxetine or saline-vehicle ~5 min before testing.

### Statistics

Behavioural data were analysed using SPSS (IBM, NY, US). All within-subject non-normalised data from each experiment (one challenge conducted for each drug condition within one cohort) was analysed once with paired *t*-tests (indicated in main figures and used for primary interpretation) and once with repeated-measures (RM) ANOVAs involving the group and the drug dose as between- and within-subject independent variables, respectively, and one of the behavioural parameters as independent variable (indicated in Supplementary Tables 2–7; Sidak-adjusted simple main-effects post-hoc tests were conducted, as appropriate), after confirmation that it met the assumptions for parametric testing (Kolmogorov–Smirnov test). Additionally, to allow bivariate correlations of CNO-induced changes, within-subject chemogenetic and pharmacological data were normalised to the corresponding value under the vehicle and the resulting (drug-value/vehicle-value) ratio was log_10_-transformed. The log-transform was conducted to ensure that increases (ratio >1) and decreases (ratio <1) are equally scaled. For the parameter that could occasionally assume the value 0% (%prematures), the actual value was added to 1 before log-transformation, throughout all analysis, to avoid distortions of the data by values <1%, which are biologically insignificant. All behavioural source data is obtainable from the corresponding author at reasonable request.

## Results

### Control of sustained attention and impulsivity by G_q_-signalling in ACC PV-interneurons

PV-Cre mice were transduced in the ACC with a Cre-dependent AAV vector expressing hM3Dq-mCherry (Gq-group) or mCherry (mCh control-group; Fig. 1b–d and Supplementary Figs. 1, 2) and trained on the 5-CSRTT until the asymptotic performance on the final baseline stage (2 s SD; 5 s ITI). They were then tested in distinct challenge protocols (Supplementary Table 1 and Supplementary Fig. 3) on separate days once per week, each after pre-treatment with CNO (0.7 or 2.1 mg/kg) or vehicle (within-subject design). Under conditions of an extended waiting time (7 s ITI), activation of G_q_-signalling in PV-interneurons reproducibly led to increased attentional accuracy—mainly due to a strong decrease of incorrect responses —and to decreased premature responding (Fig. 1e, f and Supplementary Fig. 4; see also Supplementary Table 2 for statistical details of PV-Gq experiments). These effects did not reflect a faster within-session adaptation to the extended ITI, but were present throughout the session (Supplementary Fig. 5). They were not apparent, however, when challenging only sustained attention by reduction of the SD (0.8 s) or applying the baseline protocol only, on separate test days (Fig. 1g–i; note that a selective efficacy in the ITI- as opposed to the SD challenge is only statistically supported for %prematures, not for accuracy and incorrects, when conducting a 2-way RM-ANOVA involving both challenges with the higher dose of 2.1 mg/kg CNO, Supplementary Table 3). Also, in the 0.8s-SD challenge, CNO significantly increased omission rates selectively in PV-Gq mice (Fig. 1e–i and Supplementary Tables 2, 3), which could indicate lapses of attention or task engagement in this condition. However, attentional accuracy, reward and response latencies remained consistently unaffected, indicating that the observed effects were not due to sedation, slowing of responsiveness, or reduced motivation (Fig. 1e–i).

**Fig. 2:**
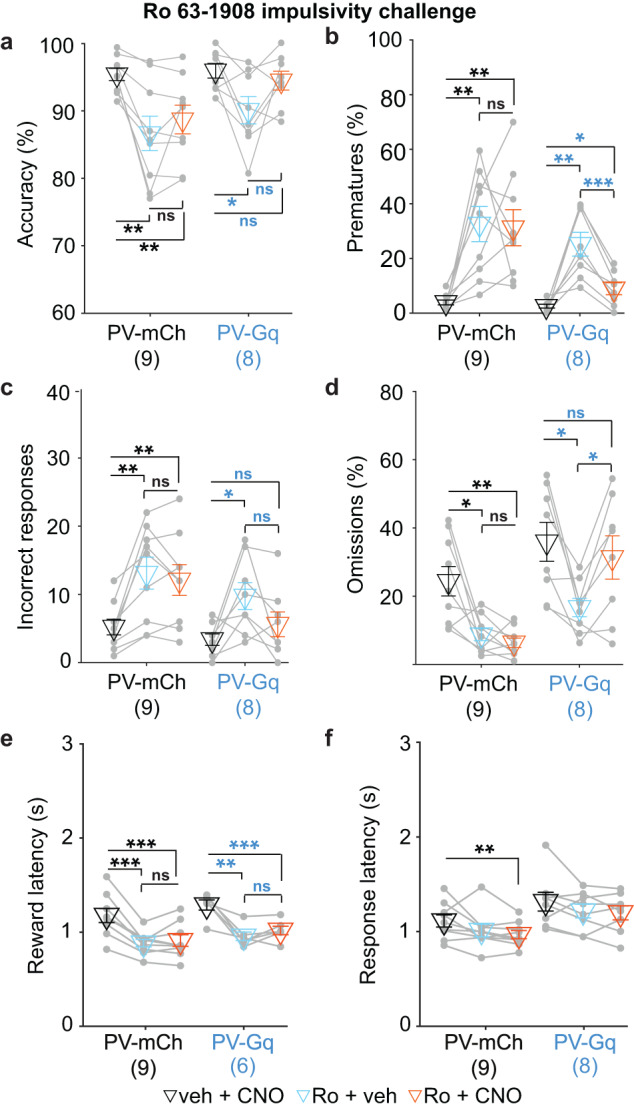
Reduction of pharmacologically induced premature responding by activation of ACC PV-interneurons. **a**–**f** Attentional accuracy (**a**), premature responding (**b**), incorrect responses (**c**), omission rates (**d**), and reward (**e**) and response latencies (**f**) after application of vehicle and CNO (black), the impulsivity-inducing compound Ro63-1908 (Ro, 3 mg/kg) and vehicle (cyan), and combined pre-treatment with Ro and 0.7 mg/kg CNO (orange) in the baseline protocol in the groups stated on the x-axes (*N*-numbers in brackets; one animal per group was excluded because Ro alone did not induce >5% premature responding; two further mice did not contribute reward latency data due to a technical error). Significance symbols indicate the results of paired *t*-test comparisons within each group. See Supplementary Table 4 for further statistics, including RM-ANOVA and post-hoc tests; see Supplementary Fig. 4 for further analysis. ^ns^ *P* > 0.05; *P < 0.05; **P < 0.01; ***P ≤ 0.001. Error bars represent s.e.m. throughout.

Mouse lines that display a combination of high waiting impulsivity and low sustained attention (i.e. ADHD-related models), which could be used to further evaluate the possible therapeutic efficacy of G_q_-signalling in ACC PV-cells, are scarce. Therefore, we adapted a pharmacological model previously used in rats [37] and established by us in mice [12]—systemic application of an antagonist of GluN2B-containing NMDA-type glutamate receptors (3 mg/kg Ro63-1908). We confirmed that Ro63-1908 increased premature responding and reduced accuracy by elevation of incorrect responses irrespective of CNO-application in the mCherry-injected control-group, while also decreasing omissions and reward latency (Fig. 2a–f and Supplementary Fig. 4). We could replicate the impulsivity-decreasing effect of PV-G_q_-signalling in this model, while numerical improvements of incorrect responses and accuracy were not significant (Fig. 2a–c). Again, omissions were increased by PV-interneuron activation, whereas the number of correct responses and reward latency remained unaltered (Fig. 2d, e). The overall pattern of changes observed across challenges and doses suggested that chemogenetic activation of ACC PV-cells led to a more considered behaviour, whereby erroneous active responses (incorrects and prematures) were selectively reduced.

### PV-interneuron-mediated and atomoxetine-induced changes to attentional accuracy are independent of omission increases

**Fig. 3:**
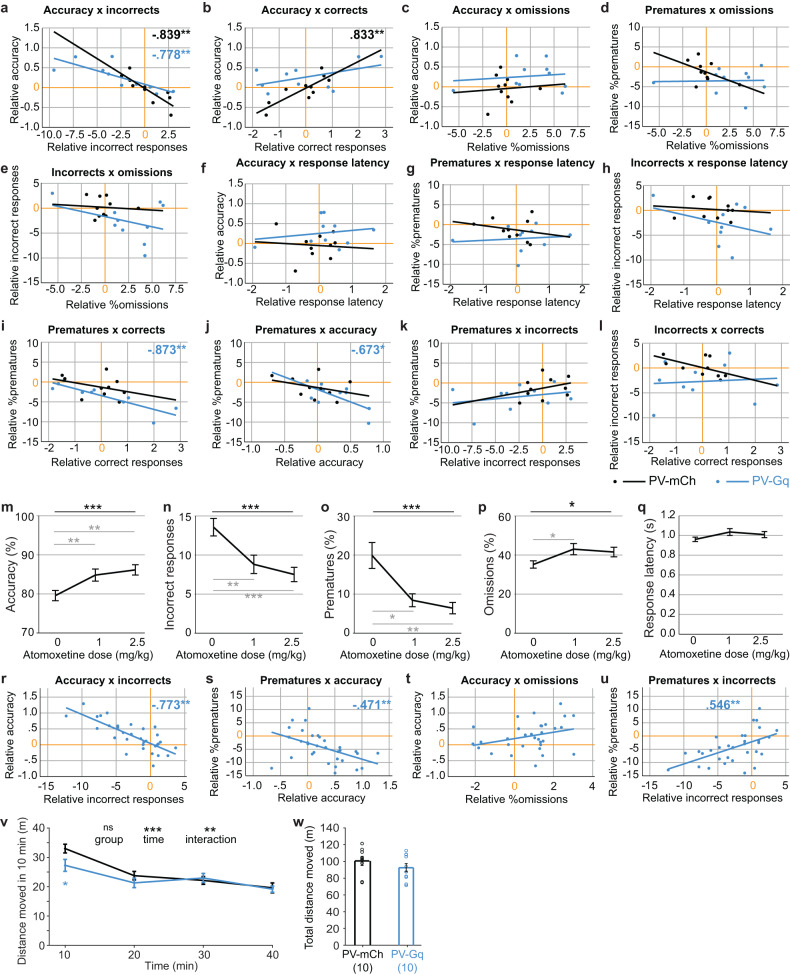
PV-interneuron-mediated reduction of incorrect and premature responses are independent from omission increases, response latency and sedation. **a**–**l** Scatter plots and group-wise linear fits illustrating bivariate correlations between log_10_-transformed ratios of the value under CNO divided by the value under the vehicle for the performance parameters of the 5-CSRTT stated on x- and y-axes (and at the top of each panel) for the 7s-ITI challenge with 2.1 mg/kg CNO. Dots represent individual mice, lines represent linear fits within each group as indicated by colour (black, mCherry-transfected controls; blue, hM3Dq-transfected mice). The orange lines indicate a lack of difference between both conditions. Where significant, correlation coefficients are stated at the top of each panel in colour corresponding to the group within which the correlation is significant. **m**–**q** Attentional accuracy, incorrect and premature responses, omission rate and response latency (from left to right, as stated on y-axes) in the 7s-ITI/0.8s-SD challenge measured after injection of saline-vehicle, 1 and 2.5 mg/kg atomoxetine (see x-axes). Results of within-subject RM-ANOVA (black) and paired Sidak post-hoc test between drug and vehicle conditions (grey) are indicated. **r**–**u** Same correlation analysis as in (**a**–**l**), but for changes induced by 1 mg/kg atomoxetine vs. vehicle. See also Supplementary Fig. 6. **v**, **w** Locomotor activity in 40 min after 2.1 mg/kg CNO shown in individual 10 min intervals assessed by RM-ANOVA (**v**) or total activity across testing time analysed by *t*-test (**w**). Significant main effects for the respective factor are stated on top of the panel (v; ^ns^ *P* > 0.05), black indicators; significant Sidak post-hoc comparison at individual intervals indicated below the data line. *P < 0.05, **P < 0.01, ***P < 0.01. Non-significant pairwise comparisons (*P* > 0.05) are not indicated. Error bars, s.e.m.

To assess, if the observed effects were interdependent, we calculated bivariate correlations between CNO-induced changes of premature responding, attentional accuracy, omissions, latencies, and the number of correct and incorrect responses for the 7s-ITI challenge with 2.1 mg/kg CNO where beneficial chemogenetic effects were most pronounced. As expected, CNO-induced changes in attentional accuracy were negatively correlated to changes in the number of incorrect responses—but not correct responses—in PV-Gq mice (Fig. 3a, b). Spontaneous fluctuations of attention in control mice, in contrast, correlated with the number of correct *and* incorrect responses (Fig. 3a, b).

Neither changes in premature or incorrect responding nor in attentional accuracy correlated with changes in omissions or response latency in PV-Gq mice (Fig. 3c–h), ruling out a general slowing down of responsiveness as a mechanism of improved impulse and attentional control. Instead, decreases in premature responses were correlated to increases in correct responses and, hence, accuracy, suggesting that the withheld premature responses are successfully translated into more correct responses (Fig. 3i, j). The reduction of premature responses (and increase in correct responses), were, however, not correlated to the reductions of incorrect responses (Fig. 3k, l), suggesting that the PV-cell-mediated effects on attention and impulse control are largely independent processes.

To further scrutinise the translational relevance of the PV-Gq-induced decrease of active erroneous responses, we assessed the effects of an approved ADHD therapeutic, atomoxetine, in the same assay in a separate cohort of wildtype mice. Surprisingly, 1 and 2.5 mg/kg atomoxetine produced a very similar profile as seen with chemogenetic activation of ACC PV-cells: attentional accuracy increased due to a decrease of incorrect responses (not due to an increase of correct responses) alongside a robust decrease of premature responses and a moderate increase of omissions—with minor and mixed effects on reward latency (Fig. 3m–q and Supplementary Fig. 6). Once again, drug-induced changes of accuracy were correlated with those of incorrect and premature responses but not with those of omissions indicating that accuracy and omissions are measuring distinct constructs (Fig. 3r–t). Of note, in contrast to the effects of the chemogenetic modulation, atomoxetine-induced decreases of prematures and incorrects were correlated with each other and, negatively, with omissions (Fig. 3u and Supplementary Fig. 6), suggesting that their reduction primarily translates into response omission rather than increased correct responding. In a separate cohort of PV-Gq mice, we found further commonalities between both manipulations at the physiological level: they both decreased high-gamma-frequency (52–80 Hz) oscillations in the dorsal posterior ACC (Supplementary Figs. 7–9).

Finally, we assessed if the chemogenetic manipulation could also reduce hyperactivity by exposing mice to a novel open-field 10 min after injection of 2.1 mg/kg CNO and monitoring locomotor activity over 40 min. There was no effect of group when analysing activity in 10 min time bins or in total (Fig. 3v, w). However, there was a significant time-group interaction (*P* = 0.009; RM-ANOVA), driven by a mildly reduced locomotion in the first time bin in PV-Gq mice compared to mCherry-controls (*P* = 0.044; Sidak post-hoc test; Fig. 3v), suggesting the efficacy of this manipulation to moderate excessive hyperactivity as induced by the initial spatial novelty in this test. A more persistent locomotion-reducing effect was seen in a separate PV-Gq cohort with simultaneous electrophysiological recordings (within-subject design: CNO vs. vehicle comparison; Supplementary Fig. 7b).

### G_i_-signalling-mediated inhibition of ACC PV-interneurons does not consistently impair 5-CSRTT performance

**Fig. 4:**
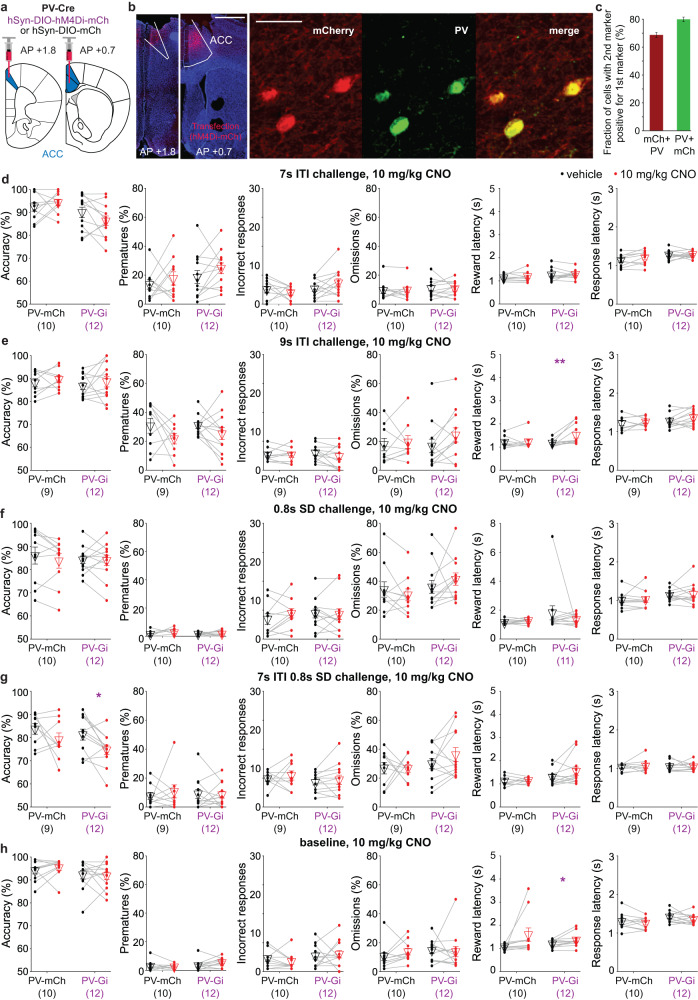
Impulsivity and attention are not consistently affected by inhibition of ACC PV-interneurons. **a** Schematic of viral transfection. **b** Example microscopic images showing expression of hM4Di-mCherry (red) in the transfected ACC region (marked by white borders hand-drawn according to slice layout and Franklin & Paxinos mouse brain atlas, 3^rd^ ed. [58]; left; scale bar, 1 mm) and expression of hM4Di-mCherry (red) and PV (green; right, scale bar, 50 μm). **c** Co-expression of mCherry in PV-positive cells (red) and the reverse (green). **d**–**h** Attentional accuracy, premature and incorrect responding, omissions and reward and response latencies (from left to right, as indicated on y-axes) in the 5-CSRTT protocol stated above each panel; i.e. the first and second ITI-challenge (**d**, **e**), 0.8s-SD challenge (**f**), the combined 7s-ITI/0.8s-SD challenge (**g**), and the baseline protocol (**h**); plotted for each individual mouse of the groups stated on the x-axes (*N*-numbers in brackets) for pretreatment with 10 mg/kg CNO (red dots) or vehicle (black dots). The order, in which the protocols were conducted in this cohort is (**d**–**h**). Purple and black symbols above the data lines indicate significant within-subject differences between vehicle and CNO pretreatment (paired *t*-test). See Supplementary Table 5 for further statistics with RM-ANOVA and post-hoc tests, and Supplementary Fig. 11 for further data from these and subsequent experiments in this cohort. *P < 0.05; **P < 0.01. Non-significant pairwise comparisons (*P* > 0.05) are not indicated. Error bars represent s.e.m. throughout.

Given the observed cognitive improvements induced by the activation of ACC PV-interneurons, we investigated, if their chemogenetic inhibition has the reverse effect on task performance [20, 28] using the G_i_-coupled receptor hM4Di. A separate PV-Cre cohort was trained in the 5-CSRTT and transfected with either hM4Di or the mCherry-control vector (Fig. 4a–c). After reaching the baseline stage, mice were taken through a testing regime with prior application of CNO (10 mg/kg) or vehicle in the previously used 7s-ITI and 0.8s-SD challenges and the baseline protocol. Surprisingly, no expected CNO-induced worsening of attentional accuracy, incorrect or premature responding—nor significant effects on omissions or response latency—were observed; only a decrease in accuracy and correct responses was detected in an additional combined 7s-ITI/0.8-SD challenge (Fig. 4d–h; Supplementary Fig. 11 and Supplementary Table 5). To reach certainty about this result, we subsequently repeated most challenges with the more potent hM4Di-agonist clozapine (CLZ), which fully confirmed the previously seen lack of consistent effects of chemogenetic PV-cell inhibition (Supplementary Fig. 11). To confirm that this constitutes a true-negative result, as opposed to a failure of DREADD-mediated manipulation, we validated that hM4Di-mediated PV-cell inhibition entails a disinhibitory effect on the local ACC circuit (Supplementary Fig. 10).

### Partially improved attentional accuracy by G_q_-signalling in ACC Sst-interneurons

**Fig. 5:**
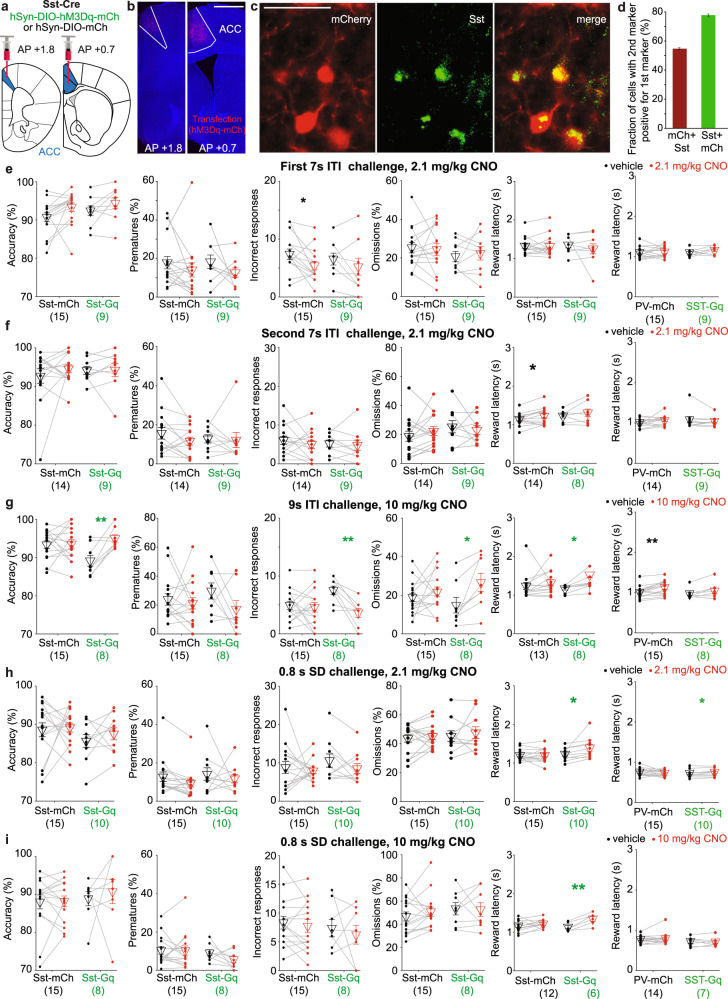
Activation of ACC Sst-interneurons partially improves attention but not impulse control. **a** Schematic of viral transfection. **b** Example microscopic images showing expression of hM3Dq-mCherry (red) in the transfected ACC region (marked by white borders hand-drawn according to slice layout and Franklin & Paxinos mouse brain atlas, 3rd ed. [58]); scale bar, 1 mm. **c** Example microscopic images showing expression of hM3Dq-mCherry (red) and somatostatin (green). Scale bar, 50 μm. **d** Co-expression of mCherry in the Sst-positive cell (red) and the reverse (green). **e**–**i** From left to right (as indicated on y-axes): Attentional accuracy, premature and incorrect responding, omissions and reward and response latencies in the 5-CSRTT protocol stated above each panel; i.e. the first and second 7s-ITI-challenge with 2.1 mg/kg CNO (**c**, **d**), the 9s-ITI challenge with 10 mg/kg CNO (**d**), the 0.8s-SD challenge with 2.1 (**f**) and 10 mg/kg CNO (**g**); plotted for each individual mouse of the groups stated on the x-axes (*N*-numbers in brackets) for pretreatment with CNO (red dots) or vehicle (black dots). The corresponding data for the baseline protocol and the numbers of correct responses are shown in Supplementary Fig. 13. The order, in which the protocols were conducted in this cohort is (**e**, **h**), baseline, (**f**, **g**, **i**). Green and black symbols above the data lines indicate significant within-subject differences between vehicle and CNO pretreatment (paired *t*-test). See Supplementary Tables 6,7 for further statistics with RM-ANOVA and post-hoc tests, and reasons for varying *N*-numbers. *P < 0.05; **P < 0.01. Non-significant pairwise comparisons (*P* > 0.05) are not indicated. Error bars represent s.e.m. throughout.

Given the absence of a behavioural effect of PV-interneuron inhibition, we hypothesised that another type of interneuron may uphold inhibitory tone under this manipulation, whereby somatostatin-positive (Sst) interneurons are the most likely candidate as they also inhibit pyramidal cells [38, 39]. This suggests that activation of Sst-interneurons may be an alternative strategy to enhance cognition. To test this hypothesis, we trained a cohort of Sst-Cre mice in the 5-CSRTT and transfected their ACC regions with hM3Dq or an mCherry-control vector (Fig. 5a–d and Supplementary Fig. 12). Using the same test protocols and initial CNO dose (2.1 mg/kg) as in the PV-Gq cohort, we found that activation of Sst-interneurons did not impact 5-CSRTT performance (Fig. 5e, h and Supplementary Fig. 13). Given this null result, we repeated the ITI-challenge with 2.1 mg/kg and both challenges with 10 mg/kg CNO (Fig. 5f, g, i). While still no changes in 5-CSRTT performance were observed in the 0.8s-SD challenge, an improvement of accuracy - via a selective reduction of incorrect responses—but not of premature responses, was detected in the 9s-ITI condition with 10 mg/kg CNO (Fig. 5g and Supplementary Table 6). Omissions also increased (Fig. 5g), and significant dose–drug interactions for accuracy, incorrects, and omissions supported the selective effect of the higher dose (*P* < 0.05, two-way RM-ANOVA across the two last ITI-challenges within the Gq-subgroup; Supplementary Table 7). In the same challenge and in the 0.8s-SD challenges, CNO also increased reward latency (Fig. 5g–i), which may indicate reduced motivation. We further assessed the effect of Sst-interneuron activation (using 2.1 mg/kg CNO) on novelty-induced hyperactivity in the open field and found a significant time-group interaction (*P* = 0.005, RM-ANOVA) driven by significantly lower activity in the first 10 min (*P* = 0.035; Sidak; Supplementary Fig. 13). In a separate Sst-Gq cohort, we found that 10 mg/kg had a robust locomotion-reducing effect across time bins (*P* < 0.05 for the effect of the drug, within-subject comparison), whereas both doses activated cFos-expression in ACC Sst-interneurons (Supplementary Fig. 14).

## Discussion

Our results show that activation of G_q_-signalling in PV-interneurons of the ACC can improve aspects of hyperactivity, motor impulsivity, and attentional accuracy in certain challenging conditions. This effect was largely specific to PV-cells, as stimulation of Sst-interneurons only showed beneficial effects in the attention and hyperactivity domains, and only at CNO doses where motivational drive appeared reduced. While the role of ACC Sst-cells needs to be followed up by future loss-of-function experiments, and also confounding effects of unilateral off-target-expression in some animals of either cohort cannot be fully excluded at this stage (see Supplementary Figs. 2, 12), the comparably limited efficacy of their activation may relate to the fact that they, in turn, inhibit PV-cells [40–42] which could offset beneficial effects of increased inhibition of pyramidal cells. Indeed, previous work in rats with local pharmacological infusions into the broader PFC area has shown that both general augmentation (with the GABA_A_-agonist muscimol) and general reduction (with the GABA_A_-antagonists bicuculline or picrotoxin) may increase omissions and decrease attentional accuracy in the 5-CSRTT [43–45], whereas muscimol may also increase premature responding [43, 44] and picrotoxin increases locomotor hyperactivity [44]. This suggests that the modulation of inhibitory neurons needs to be rather *precise* in terms of affected cell type or synapse and strength (dosing) in order to be therapeutically effective. This is also exemplified by the finding that low frequency (1–10 Hz) optogenetic stimulation of dorsal PFC PV-cells increased premature responding [20], which contrasts with the opposite effect seen here with hM3Dq-activation. Likewise, whereas such slow stimulation also increased omissions (as chemogenetic modulation in our hands did), 30–40 Hz stimulation had the opposite effect [20]. These observations exemplify the importance of the appropriate activation pattern for therapeutic efficacy; for example, optogenetic stimulation strongly synchronises the activity of a given cell population and enforces an external rhythm, while chemogenetic stimulation rather increases excitability, possibly leading to the enhancement of endogenous activity.

As a reduction of incorrect and premature responses was not seen with optogenetic activation of PV-cells in the broader dPFC area [20], nor here with the 0.8s-SD challenge, this effect likely requires impulse control to be challenged to be detected. From a translational perspective, such a strong efficacy in states of behavioural challenges and weak modulation in unchallenged states is actually desired. However, this response pattern also makes plausible an alternative interpretation of our data, namely that—rather than impulse control—it is the adaptation of the temporal strategy that mice deploy during the task that is improved [46]. In this scenario, mice adapt to the fixed 5s-ITI during task acquisition [46], and this rhythm is disrupted by the fixed 7s-ITI challenge requiring fast within-session adaptation. Our within session-analysis, however, refutes this scenario, as premature responding was already reduced at the beginning of the session in CNO-treated PV-Gq mice and showed no further reduction (i.e. adaptation; Supplementary Fig. 5). Furthermore, mice do not rely strongly on a temporal strategy to solve the 5-CSRTT [46] and temporal adaption cannot easily explain the profound decrease of incorrect responses (which are made only after waiting throughout the ITI and which are not correlated to decreased premature responding) nor the efficacy against increased premature responding in the Ro-challenge in which the ITI is unaltered.

A further potential caveat of our conclusion is that PV-Gq activation led to increases in omissions in the 0.8s-SD challenge (whereas numerical omission increases in the 7s-ITI challenge, where therapeutic effects were detected, were not significant). Given the lack of concomitant within-subject increases of reward and response latencies, such omission increases are not an indicator of reduced responsiveness (sedation). Rather, this profile suggests that the chemogenetic modulation could evoke either a passive failure or an active restraint to respond at all when the subject is unsure of the correct response, at least under certain conditions when attentional demand is very high. This would also align with its lack of an effect on accuracy in the same challenge. Omissions have been considered as an additional indicator of inattention in the 5-CSRTT by some researchers [20, 47, 48], which would question the overall beneficial effect of PV-cell activation on attention concluded here. However, given that the primary indicator of attention—accuracy [17]—does improve in the 7s-ITI challenge and that changes in response latency are not present and uncorrelated to accuracy improvements (with the dose and protocol where accuracy and impulsivity improved most robustly), an increase of inattention is not a plausible interpretation of the chemogenetically induced behavioural profile. The lack of a correlation between omission increases and changes to accuracy, incorrect and premature responses at the level of individual mice, also argues against mediation of the observed performance improvements by omission increases. Finally, it has been remarked that pharmacologically induced omission increases seen in rodents, e.g. with guanfacine or atomoxetine, do not translate to inattention in humans [49]. This conclusion has been further validated by our correlation analysis of atomoxetine-induced changes in the 5-CSRTT, which were strikingly similar to those seen by activation of ACC PV-interneurons. Therefore, while omissions *may* be driven by inattention under some circumstances, they are not necessarily an indicator of it on their own, and they do not predict a lack of therapeutic efficacy in ADHD. The overall response profile of PV-cell activation seen here is more consistent with the conclusion that the additional omissions in the 0.8s-SD challenge represent a selective, active choice to not respond in cases of uncertainty which is rather independent of the decreases of incorrect and premature responses seen in the 7s-ITI challenge. Nevertheless, we cannot rule out the alternative explanation that omissions reflect another aspect of attention that is independent of that represented by accuracy, and that is worsened by ACC PV-interneuron activation and atomoxetine treatment.

At a physiological level, excitatory neurons in rodent ACC represent chosen actions and carry information about predicted consequences [50, 51]. Therefore, elevated inhibitory tone may suppress signals associated with action selection, increasing the evidence required to commit to action, and hence accuracy and impulse control. A recent optogenetic study showed that optogenetic gamma-range stimulation of ACC neurons projecting to the visual cortex may reduce incorrect responding [52]—and thus, chemogenetic activation of a large proportion of ACC PV-cells that innervate these neurons, conferring gamma-rhythmic activity onto them [53], may underlie the improvement of accuracy. Chemogenetic inhibition of the same neurons (possibly mimicked by strong activation of inhibitory neurons), in turn, may increase omissions [54]. In reverse, as we recently showed that chemogenetic reduction of the activity of layer 5 pyramidal cells of the ACC may reduce premature responding—with virtually no effect on accuracy or omissions – the activation of PV-cells inhibiting these layer-5 projection neurons may rather be relevant for the impulsivity-decreasing effect [12]. Further physiological investigation is required to illuminate the mechanistic underpinnings of the differential behavioural effects seen here with chemogenetic modulation.

In conclusion, the present data is the so far most direct demonstration that activation of GABAergic inhibition in the ACC may indeed be a viable therapeutic concept in impulse control disorders, and narrows it down to the enhancement of PV-interneurons, rather than GABAergic signalling in general. In contrast to the alternative strategy of decreasing the excitation of pyramidal cells directly—which has shown differing effects on attentional accuracy in different studies (likely reflecting targeting of different populations of excitatory cells) [12, 55]—an activation of PV-cells may also benefit sustained attention. Hence, our data points to G_q_PCRs in ACC PV-cells as potential targets for treating core symptoms of ADHD. Large-scale mouse and human datasets of gene expression in the different ACC cell-types exist [56], that allows the search for suitable GPCR targets expressed endogenously with relative specificity in ACC PV-interneurons [12]. The findings that hM3Dq-activation in prelimbic or hippocampal PV-interneurons improves sociability [28] or associative fear-learning [57], respectively, in preclinical psychiatric disease models, further support the conclusion that agonists of G_q_PCRs expressed in PV-cells may be sufficient for achieving therapeutic efficacy in various psychiatric impairments (instead of requiring gamma-frequency stimulation). Therefore, a temporally continuous, rather than frequency-modulated, increase of ACC PV-neuron excitability induced pharmacologically through G_q_PCRs may be a viable strategy to improve aspects of impulsivity, hyperactivity, and sustained attention. Our results also highlight the importance of assessing multiple challenge conditions to characterise cognitive enhancement.

## Supplementary information

Supplementary Information

## Data Availability

All source data can be obtained from the corresponding author upon reasonable request. Task protocol files are available from https://github.com/KaetzelLab/Operant-Box-Code.
