# Supplementary material for: Control of sustained attention and impulsivity by Gq-protein signalling in parvalbumin interneurons of the anterior cingulate cortex
Source: Transl Psychiatry. 2023 Jul 5;13:243. doi: 10.1038/s41398-023-02541-z (PMC10323001; doi:10.1038/s41398-023-02541-z)
Supplement: Supplementary file 1 — Supplementary Information [file 41398_2023_2541_MOESM1_ESM.pdf]

Supplemental Information

Control of sustained attention and impulsivity by G_q-protein signaling in parvalbumin interneurons of the anterior cingulate cortex

Key words: impulsivity, sustained attention, parvalbumin, anterior cingulate cortex, G-protein-coupled receptors, chemogenetics, ADHD

Martin M. Jendryka [1,2], Uwe Lewin [1], Bastiaan van der Veen [1], Sampath K.T. Kapanaiah [1], Vivien Prex [1], Daniel Strahnen [1], Thomas Akam[3], Birgit Liss[1,4], Anton Pekcec [2,5], Wiebke Nissen [2,5], Dennis Kätzel [1,5,6]

[1] Institute of Applied Physiology, Ulm University, Ulm, Germany

[2] Boehringer Ingelheim Pharma GmbH & Co. KG, Div. Research Germany, Biberach an der Riss, Germany

[3] Department of Experimental Psychology, University of Oxford, Oxford, UK

[4] Linacre College and New College, University of Oxford, Oxford, UK

[5] These authors contributed equally to this work

[6] Correspondence: dennis.kaetzel@uni-ulm.de; +49 731 500 33770; Fax +49 731 500 33779; Institute of Applied Physiology, Ulm University, Albert-Einstein-Allee 11, 89081 Ulm, Germany

Supplementary Methods

Surgery for viral transduction

Once mice had reached at least stage 3 of 5-choice-serial-reaction-time task training (see below), they were assigned to the control or the DREADD group, based on their performance over the first 3 days of this stage as a measure of counter-balancing. Animals were anaesthetized using isoflurane (AbbVie, DE), received s.c. injections of analgesics (0.08 mg/kg buprenorphine, Bayer, DE; 1 mg/kg meloxicam, Boehringer Ingelheim, DE), and local scalp anaesthesia (200 μl of 0.025 % bupivacaine, AstraZeneca, UK) before placement in a stereotaxic frame (motorized and atlas-integrated frame, Neurostar, DE, and Kopf, US; manual digital frame, World Precision Instruments, US or Kopf, US) with non-rupture mouse ear bars. The body temperature was stabilized using a feedback-controlled heating blanket (Harvard Apparatus, US) and the anaesthesia was maintained with ~1.5 % isoflurane. The following stereotaxic coordinates (from bregma) and volumes were used for bilateral transfection of the ACC: posterior injection at AP +0.7, ML 0.3, DV 1.65 (200 nl) and 1.3 (300 nl), anterior injection at AP 1.8, ML 0.25, DV 1.25 (80 nl). All viral vectors were of serotype AAV8 and were obtained from the University of North Carolina vector core (UNC, NC, US; hM3Dq) or the University of Zürich viral vector facility (UZH-VVF, CH; hM4Di, mCherry). Suspensions of the hM3Dq-mCherry and mCherry-control virus were diluted down to a final titre of $2.9*10^{12}$ vg/ml in 5 % sorbitol/PBS (Sigma, DE), hM4Di-mCherry-AAV and its mCherry-control virus were used at $5.0*10^{12}$ vg/ml. All *Cre*-dependent vectors were equal in backbone sequence, differed only with respect to its DREADD-mCherry fusion insert and were based on the pAAV2-hSyn-DIO-DREADD-mCherry-WPRE-hGHpA Addgene constructs 44361 (hM3Dq), 44362 (hM4Di), and 50459 (mCherry) from the Bryan Roth laboratory (https://www.addgene.org/Bryan_Roth/). A pouch to absorb the virus was created by moving the needle 0.05 mm further down and then up again to the actual DV position before infusion. Infusions were made using a glass 10 μl precision syringe (WPI or Hamilton, US) at an injection rate of 50 or 100 nl/min. Mice received post-operative monitoring for 7 d, and the analgesic meloxicam (Metacam, 1 mg/kg, Boehringer Ingelheim, DE) for 3 days. The mice were kept on *ad libitum* food for a minimum of two weeks before training in the 5-CSRTT commenced.

Surgery with chronic electrophysiological implantations and awake-state recordings during the 5-CSRTT and LMA testing

Implantation surgeries were timed and conducted like normal surgeries for viral transduction (see above), except that 6 single polyimide-insulated tungsten wires of 50 µm diameter

(WireTronic Inc., CA, US) and two surface electrodes (scull screws; 1.2 mm diameter, Precision Technologies, UK) were implanted as chronic field electrodes immediately after the last AAV infusion into the ACC (Cg1/2). Coordinates for the various regions were as follows with reference to Bregma (in mm) and DV measured from pia: Cg1-anterior (Cg1a; AP +1.8-1.9, ML 0.35; DV 0.6), prelimbic cortex (PrL; AP +1.8-1.9, ML 0.35; DV 1.8), Cg1-posterior (Cg1p; AP +0.6, ML 0.3; DV 0.6), Cg2 (AP +0.6, ML 0.3; DV 0.6), dorsal hippocampus (dHC; AP -2, ML 1.5, DV 1.4), primary visual cortex (V1; AP -3.8, ML 2.9, DV 0.5), frontal cortex (FC; AP +4, ML -1, DV 0; screw), ground (AP -5.5, ML -1; DV 0; screw). Tungsten electrodes were implanted in the right, screws above the left hemisphere. All electrode wires were connected to gold pins in a dual-row 8-pin connector (Mill-Max, UK). Tungsten electrode placements were determined *post-mortem* from electrolytic lesions made under terminal ketamine/medetomidine anaesthesia followed by perfusion-fixation. Misplaced electrodes were excluded, leaving the following number of used electrodes per region for the 9 hM3Dq- and the 5 mCherry-transduced implanted mice: Cg1a (8/5), PrL (8/5), Cg1p (9/5), Cg2 (9/5), dHC (9/5), V1 (7/5), FC (9/5).

After recovery from surgery, training was continued in the 5-CSRTT until the final baseline stage (5). Within-subject chemogenetic and pharmacological experiments were conducted just like in the other cohorts (see main Methods and Behavioural testing below), albeit with a mounted and tethered headstage for simultaneous recording of LFP signals. Experiments were conducted in the same custom-made pyControl-based 5-choice operant boxes (pyOS-5) as the behavioural testing in the main PV-Gq and Sst-Gq cohorts was done because these boxes and their control hardware and software are optimized for electrophysiological recordings as described in detail elsewhere [1,2]. For recording, a 32-channel RHD2132 digitizing headstage (Intan, US) connected to the implaned Mill-Max connector through a custom-made Mill-Max-to-Omnetics (A79022-001, MSA components, G) adapter was used. To allow continuous recordings in operant boxes and in the open-field, a custom-made motorized commutator was used that could sense the torque on the 12-channel ultra-thin SPI-cable (Intan) created by horizontal rotations of the mouse and supported the turning of the cable through a motor to prevent winding-up. LFP-data and simultaneous TTL-signals indicating behavioural events generated by pyControl/pyOS-5 (time-stamps) were acquired with the *Open-EPhys* acquisition board and software at a sampling rate of 20 kHz; whereby both unfiltered and bandpass-filtered (0.1-300 Hz) LFP data were recorded simultaneously, and the latter was used for power analysis.

Surgery for tetrode-recordings under anaesthesia

Male PV-Cre mice that had undergone surgery for viral transduction (see above) and were transduced with either an hM4Di-expressing AAV (*N* = 5) or no AAV (*N* = 3) in the ACC. They were used for tetrode recordings under terminal anaesthesia several months later. The mice were anaesthetized with isoflurane as described above (see Surgery) to temporally implant a microdrive (Axona Ltd., UK) holding four movable tetrodes for extracellular recordings, whereby two tetrodes were placed in each hemisphere. One tetrode was constructed from four 12 µm-diameter tungsten wires (California FineWire Company, US), which were twisted and fused together by heating the insulation. The tetrodes were held in a microdrive assembly (Axona Ltd) that allowed them to be lowered or raised individually. The impedance of the tetrodes was reduced to 300-500 kΩ by gold-plating of the wire tips before implantation. The craniotomy was made bilaterally either over the anterior (AP +1.8, ML 0.25) or the posterior part of the ACC (AP +0.7, ML 0.3). A stainless-steel screw was implanted into the bone above the cerebellum serving as reference and ground signals. The dura mater above the regions of interest was removed, and the tetrodes were inserted at the centre of the craniotomies down to 1.3 mm DV into the brain. The ground wires of the microdrive were connected to the ground screw.

The recording was started when the animal was stably breathing without any signs of pain perception at an isoflurane concentration of 0.8-1.0% and spiking activity was visible at the chosen recording site in Cg1. Firstly, a baseline of approx. 10 min was recorded, followed by an i.v. injection of saline vehicle into the tail vein and a further 10 min of recording. Subsequently, 10 mg/kg CNO was applied i.v. and the signal was recorded for another 120 min (or shorter in one control mouse). The neural signals were recorded using the Omniplex Neural Data Acquisition System (Plexon Inc., US) at a sampling rate of 40 kHz and a gain of 5000.

Behavioural testing

Behavioural testing was done blind to the subgroup identity of the mice. Mice started training in the **5-choice-serial-reaction-time task** (5-CSRTT) at 2-3 months of age and were kept under food-restriction at 85-95% of their average free-feeding weight which was measured over 3 days immediately prior to the start of food-restriction at the start of the behavioural training. Training was conducted on 5-7 days per week and lasted 5, 8, and 12 weeks for the SST-Gq, PV-Gq, and PV-Gi cohorts, respectively, excluding intermittent surgery and recovery time. Water was available *ad libitum*.

5-CSRTT training and testing was conducted in operant chambers placed individually in melamine-MDF sound-insulated and ventilated cubicles. For logistic reasons, the PV-Gi

cohort and the WT cohort for atomoxetine experiments were trained and tested in a separate set-up that contained touchscreen-based 5-choice mouse operant chambers (Campden Instruments, Cambridge, GB) while custom-built operant boxes with classical poke-holes were used for the hM3Dq-cohorts. In both cases, the basic layout of the box was of trapezoidal shape and the recesses of the 5-choice wall were similarly spaced; IR break-beams detected entry and exit into all recesses and the reward receptacle. All apertures could be illuminated to instruct the entry into them.

After initiation of food-restriction to maintain animals at 85-95 % free-fed baseline weight, mice were accustomed to consume the reward (strawberry milk, Müllermilch™, DE) first in their home cage, and then in the operant box (2-3 exposures each). Subsequently, mice were trained in 8-12 sessions (30 min, once daily) of *habituation training*. Here, all holes of the 5-choice wall were illuminated for an unlimited time and the mouse could poke into any one of them to earn a 40 µl milk reward subsequently disposed from the illuminated receptacle. If mice attained at least 30 rewards each in two consecutive sessions, they were moved to the *5-CSRTT training*, during which mice transitioned through five stages of increasing difficulty, based on reaching certain performance criteria in each stage in a single session [3,4]; see Supplementary Table 1 for parameters defining all training and challenge stages including transition criteria. On each stage, certain minimal performance criteria in terms of high accuracy and response rates had to be achieved in one session in order to transition to the next stage. Given that mice generally displayed lower omission rates in the touch-screen chambers, the transition criteria were adjusted for the PV-Gi and the WT cohorts that were trained in those boxes (see [4]), demanding ≥60 correct responses (instead of ≥40) and ≤30 % omissions (instead of ≤40 %) in the last three training stages, while the accuracy criterion was identical for all cohorts (≥80 %). Also, the first 2 training stages were merged in these cohorts and combined challenges with reduced SD (0.8s) alongside increased ITI were partly used for testing (as stated in figure legends) to avoid ceiling of accuracy values. The difficulty of the stages was determined by the length of time the stimulus was presented (stimulus duration, SD) and the length of waiting time between the end of the previous trial and the stimulus presentation of the next trial (inter-trial-interval, ITI). In case a reward was collected on the previous trial, the ITI was initiated by the removal of the snout of the animal from the reward receptacle. In all 5-CSRTT protocols (Figure 1a) only one pseudo-randomly selected aperture of the 5-choice wall was lit up after the ITI, indicating that this hole needs to be poked into (correct response) in order to earn a 20 µl milk reward. If the animals either poked into any hole during the ITI (premature response), poked into a non-illuminated hole (incorrect response) during the SD or limited-hold time (LH, until 2 s after SD), or failed to poke throughout the trial (omission), trials were not rewarded but instead terminated immediately

with a 4 s or 5 s time-out period during which the house light was turned off. The relative numbers of such response types were used as performance indicators measuring premature responding [%premature = 100*(number of premature responses)/(number of trials)], sustained attention [attentional accuracy = 100*(number of correct responses)/(number of correct and incorrect responses combined)], and lack of participation [%omissions = 100*(number of omissions)/(number of correct, incorrect and omitted responses)]. Also, the time required to poke into the indicated hole after it was illuminated (response latency) and the time from the exit from the correct hole until the entry into the reward receptacle (reward latency) were measured, whereby the latter is usually used as a compound indicator of motivation and locomotor drive [5]. In some rare cases, average reward latency values of individual mice had to be excluded from the datasets because they were unrealistically low (< 0.3 s) or high (> 10 s) due to a technical error of detecting receptacle entry times (break-beams getting partially occluded by milk-reward). In almost all stages and tests, sessions lasted 30 min and were performed once daily at the same time of day and in the same box for each animal. Sessions for the testing of effects of atomoxetine vs. vehicle in the WT cohort lasted 60 min. 9 hM3Dq-transduced and 5 mCherry-transduced PV-Cre mice, each implanted with chronic field electrodes, were trained and tested in the same way, albeit with simultaneous recordings through a tethered head-stage which was connected immediately before the start of testing. Some mice did not contribute data to individual experiments for various reasons stated in the legend of the respective Supplementary Table. All operant task scripts applied in Gq-cohorts are available from https://github.com/KaetzelLab/Operant-Box-Code.
Chemogenetic and pharmacological experiments in the 5-CSRTT were mostly followed by 6-7 wash-out days during which training was conducted on at least 2-3 days, including the days immediately before testing.

For measurement of **locomotor activity** (LMA) mice of the main Sst-Gq and PV-Gq cohorts were placed into a novel clear plastic cage (425 x 266 x 185 mm; Eurostandard Typ III, Tecniplast, IT/DE) filled with clean sawdust 15 min after application of 2.1 mg/kg CNO (between-subject design) and left to explore for 40 minutes. CCTV cameras (Sentient, UK) installed centrally above the open-field cage were used to monitor each animal. Video-recordings from eight cage-stations were assembled into a single image frame through a CCTV-system (Dahua Inc, CN), digitized through an A/D converter (TheImagingSource, DE), and fed into ANY-maze (San Diego Instruments, US), for video-tracking of movement. The total distance travelled was extracted from ANY-maze for the whole time of the experiment. In an additional 9 hM3Dq-transduced Sst-Cre mice, the test was conducted in the same way, except that a within-subject design was used, i.e. three repetitions were conducted 5-6 d apart and either vehicle, 2.1 or 10 mg/kg CNO (counter-balanced across the three test days) were

injected 15 min before the start of the 40 min run (see Supplementary Fig. 14). In an additional 9 hM3Dq-transduced and 5 mCherry-transduced PV-Cre mice, each implanted with chronic field electrodes, the LMA-test was slightly modified: mice were connected to the tethered headstage and explored the novel open field for 10 min. Subsequently, either 2.1 mg/kg CNO or vehicle (within-subject design; repetitions conducted 4-5 d apart) were injected and the LMA-testing and simultaneous recording were continued for another 50 min (see Supplementary Fig. 7).

Histology

After behavioural testing was completed, animals were given an over-dose of ketamine/medetomidine (≥200 mg/kg ketamine, Zoetis, G; ≥2mg/kg medetomidine, Pfizer, US) and perfused with 0.01 M phosphate-buffered saline (PBS) followed by 4 % PFA/PBS. Additionally, for analysis of cFos-expression induced by hM3Dq-activation in Sst-Cre mice, animals were injected i.p. with either vehicle, 2 or 10 mg/kg CNO approx. 90 min before terminal ketamine/medetomidine anaesthesia. The brains were rapidly removed and then stored in 4 % PFA/PBS overnight before placement in 20 % sucrose for dehydration before sections were cut at 60 μm thickness on a vibratome (VT1000, Leica, DE). Every second section was stained with DAPI (10^{-4} % w/v) for 30 min, washed with PBS twice and mounted on glass slides. A Leica DM6B epifluorescence microscope (Leica, DE) was used to scan the slides with a 5x objective and determine virus expression. Animals were only included in the datasets if they showed bilateral expression in the majority of the volume of the target structure but no bilateral expression in the majority of the volume of any other brain region. Brains from animals with additional chronic LFP-electrodes were sliced around the region of electrode implantations, in addition to the ACC, with a slice thickness of 80 μm to identify the location of electrodes from lesion sites.

Immunohistochemistry

For the determination of co-expression of parvalbumin or somatostatin with DREADD-mCherry, brain sections were washed three times in PBS and incubated for 20 min in PBS/ 0.5% Triton™-X100 (Sigma Aldrich, USA) for permeabilization, followed by blocking with PBS/ 0.3% Triton™-X100/ 20% normal goat serum (NGS) for 60 min under gentle agitation. After washing in PBS, brain sections were incubated with the primary antibody (1:1000) in PBS/ 0.3% Triton-X100/ 1% NGS at 4°C for two days. Primary antibodies were rabbit anti-PV (Swant, CH) or rabbit anti-SST (BMA Biomedicals, CH). For secondary antibody staining, sections were washed in PBS for 10 min and afterwards incubated with goat anti-Rabbit-A488

(1:1000, Thermo Fisher Scientific, US) in PBS/ 0.3% Triton™-X100/ 1% NGS for 2 h in the dark followed by washing with PBS for 10 min. Finally, brain sections were stained with DAPI, mounted on object slides and stored at 4°C until further use. Images were taken using the Leica DM6B epifluorescence microscope with a 20x objective. The co-localization of the DREADD marker mCherry and the PV or SST antibody was quantified manually using the Image J distribution Fiji [6]. For the determination of cFos-expression in hM3Dq-mCherry-positive and –negative ACC cells in Sst-Cre mice, a similar protocol was used, except for the following deviations: blocking solution contained 0.3% Triton™-X100 and 5% NGS in PBS, carrier solution for the primary antibody contained 1% normal horse serum (NHS) and a polyclonal rabbit anti-cFos antibody (9F6; Cell Signaling Technology #2250; 1:1000) in addition to 0.3% Triton in PBS, and the carrier solution for the secondary antibody contained 1% NHS and 0.1% Triton in PBS. Images were acquired with a 5x objective at a Leica DM6 epifluorescence microscope. Counting was done blind to prior treatment.

Electrophysiological analysis

Analysis of tetrode recordings in PV-hM4Di mice

The recorded signal was high-pass filtered with a 4-pole Butterworth filter and a cut-off frequency of 400 Hz to receive the multi-unit spiking activity (MUA). Peaks were detected as spikes when crossing a negative threshold of four standard deviations below the mean signal of the total recording time. The number of spikes was analysed in 10 min bins consisting of the first (baseline) bin, the second bin (activity after vehicle injection) and 10-12 further bins following CNO-injection.

Analysis of LFP recordings in PV-Gq mice

Local power of field potential oscillations was computed from LFP recordings as described by us before for the data from the LMA test [7] and for operant testing [8], respectively, using MatLab (MathWorks, US). In brief, data – that had been band-pass filtered during acquisition between 0.1-300 Hz using *Open-EPhys* – was downsampled from 20 to 1 kHz, detrended using the *locdetrend* function of the Chronux toolbox (http://chronux.org/)) with 1 s of data and a sliding window of 0.5 s. Electrodes that were misplaced according to lesion-sites were excluded. For data from the 5-CSRTT, computations were done across all available trials, and trials were excluded from further analyses, if the amplitude exceeded the 5th standard deviation within each channel for more than 10% of the trial duration. Power spectra were calculated with routines implemented in the Chronux toolbox using the multi-taper method [9]. Power values

were expressed as $10*\log_{10}$ values and were calculated for the range of 0.1-80 Hz. For LMA-data, a bandwidth of 0.2 Hz and a total of 220 tapers were used to calculate power over the course of each of the six 10-min bins of the experiment and further statistical analysis was conducted on the average power in each frequency range and time-bin. For the analysis of 5-CSRTT data, a time-bandwidth product of 9 and 17 tapers were used to calculate power during defined time-periods – from 5 s before until 3 s after a response or omission - of each trial, whereby trials were sorted by response type (correct, incorrect, premature, omission) within each session and animal before, and such original trials were further divided into 99% overlapping “pseudo trials” with a length of 600 ms and padded to the next power of two within each category. In this way, the average power was calculated for each response type, session and animal in every frequency band. In all analysis, the following frequency bands were used: delta (δ, 1-4 Hz), theta (θ, 5-12 Hz), beta (β, 15-29 Hz), low-gamma (γ,l, 30-48 Hz), and high-gamma (γ,h, 52-80 Hz). The 48-52 Hz range was spared to avoid potential confound by 50 Hz noise, for all analyses. Mean power was calculated by averaging values across the power spectrum within the given frequency band; for theta, the maximum power was calculated, in addition (and used for the analysis of the 5-CSRTT data) by extracting the maximum average power value in the theta-frequency range.

Supplementary Figures

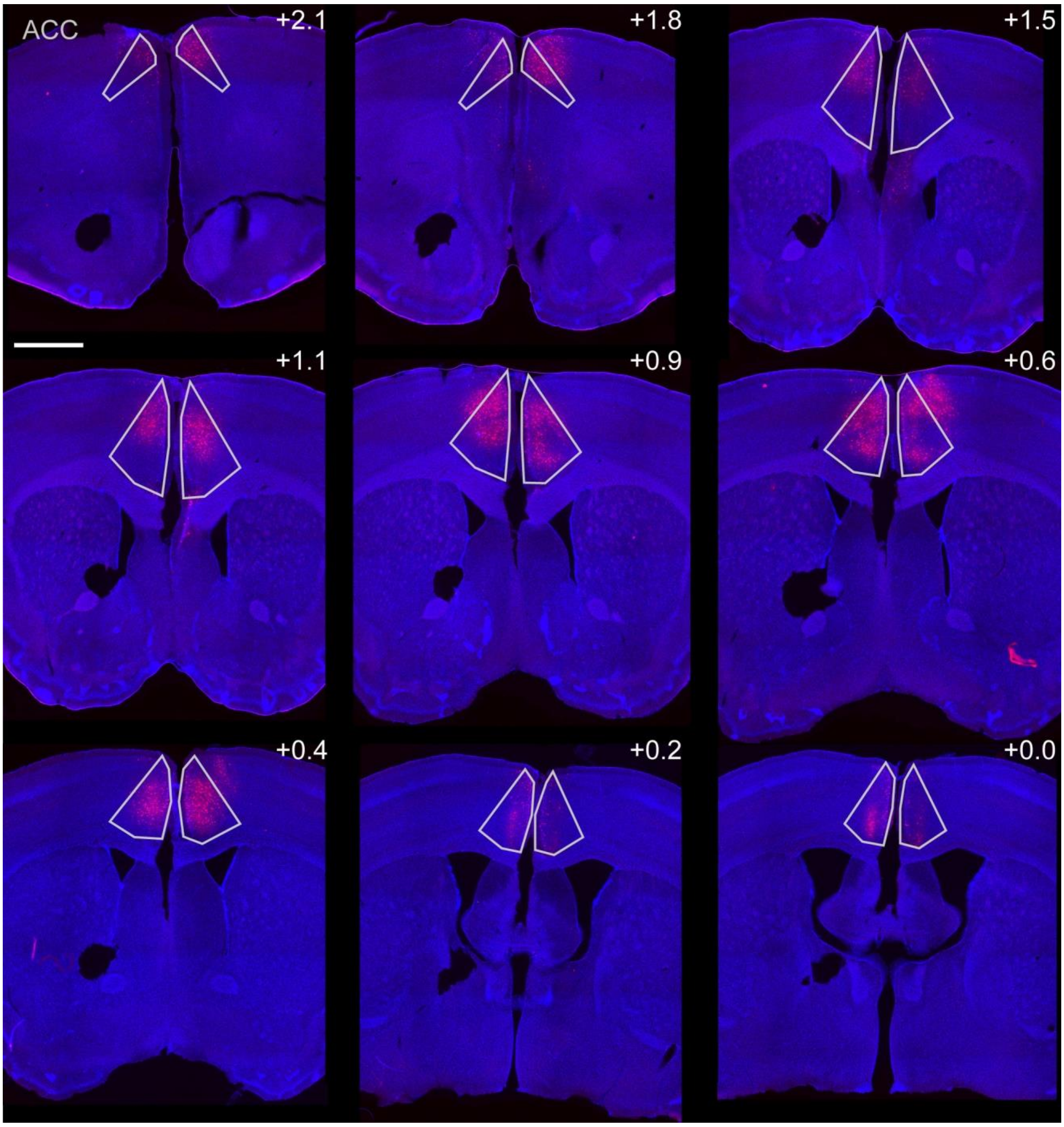

Supplementary Figure 1. Targeting of ACC PV interneurons. Cre-dependent expression of hM3Dq-mCherry (red) in PV-interneurons (PV-Gq cohort) of the ACC (approximate location marked by white borders hand-drawn according to slice layout and Franklin & Paxinos mouse brain atlas, 3rd ed.[12]) in slices from the same mouse assorted in anterior-posterior order (approximate AP coordinate stated on the top right of the corresponding slice). DAPI-counterstain in blue. Scale bar, 1 mm.

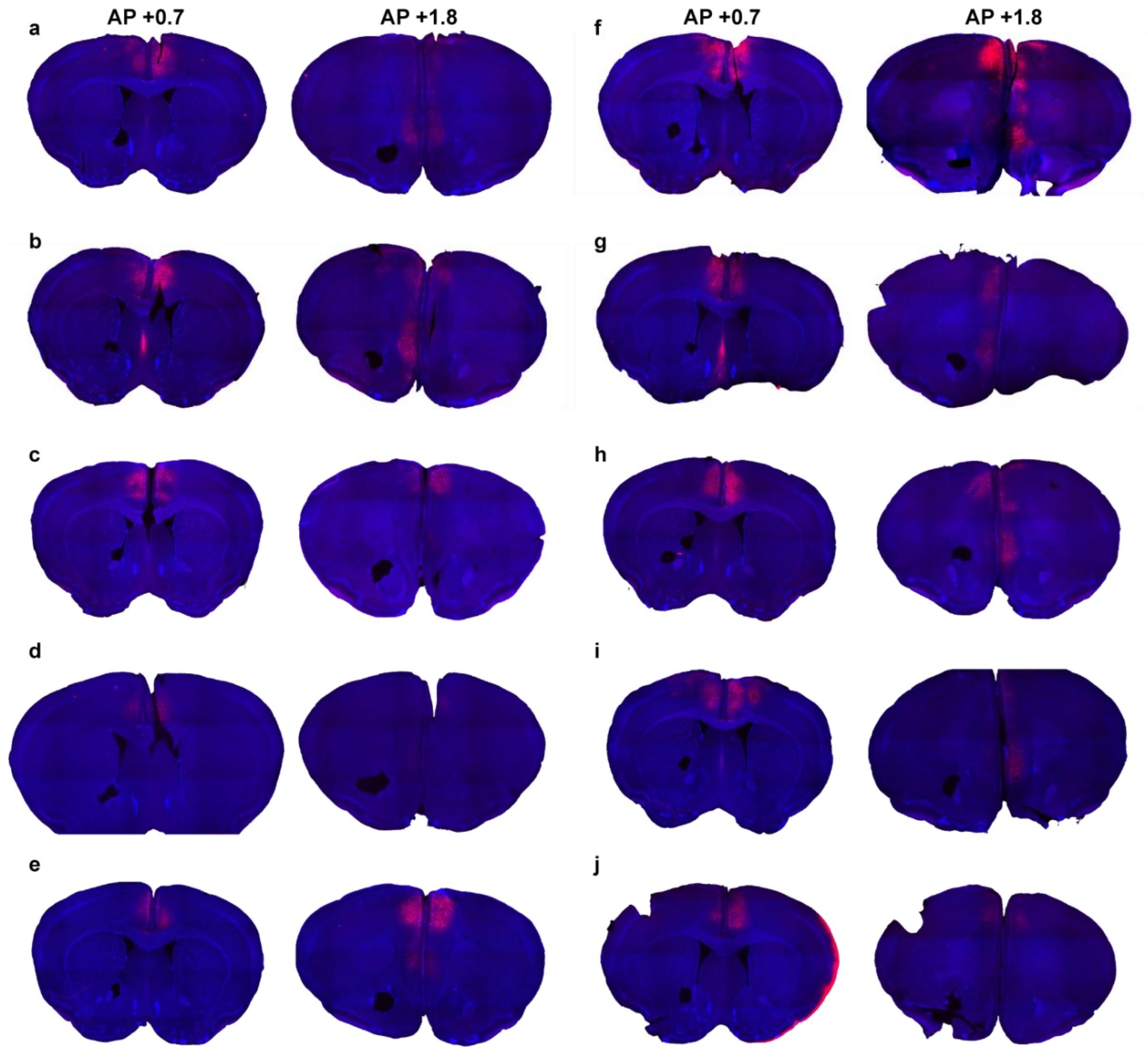

Supplementary Figure 2. Targeting of ACC PV interneurons with hM3Dq across animals. hM3Dq-mCherry expression in every mouse (a-j) of the PV-Gq cohort at the approximate anterior-posterior (AP) sites of transfection.

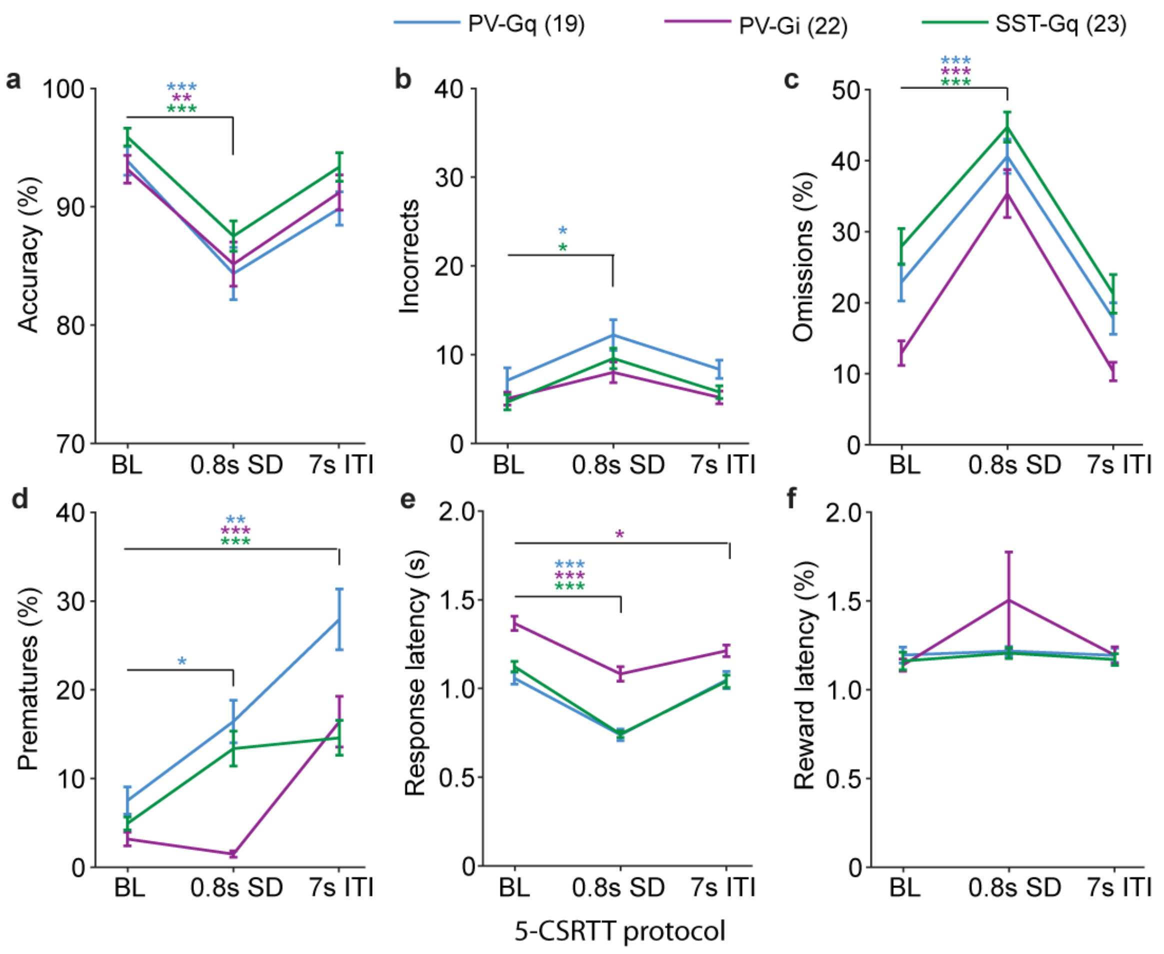

Supplementary Figure 3. Effect of parametric behavioural challenge conditions. (**a-f**) Average performance parameters (as indicated on y-axes, a-f) in the first and primary 5-CSRT-task protocols for challenging sustained attention (0.8s SD) and impulse control (7s ITI) compared to the baseline condition (BL; 2s SD, 5s ITI). Data obtained from the performance after vehicle injection in each of the three main cohorts (coded by colour above top panels with N-numbers in brackets, vehicle data from DREADD- and mCherry transfected mice of each cohort were merged). Paired Tukey-Kramer post-hoc tests between BL and each of the challenge conditions were conducted within each cohort after a significant main effect of challenge (RM-AMOVA) and are indicated by asterisks at the top of each graph coded by the colour of each cohort. Non-significant pairwise comparisons (*P* > 0.05) not indicated. Error bars, s.e.m.

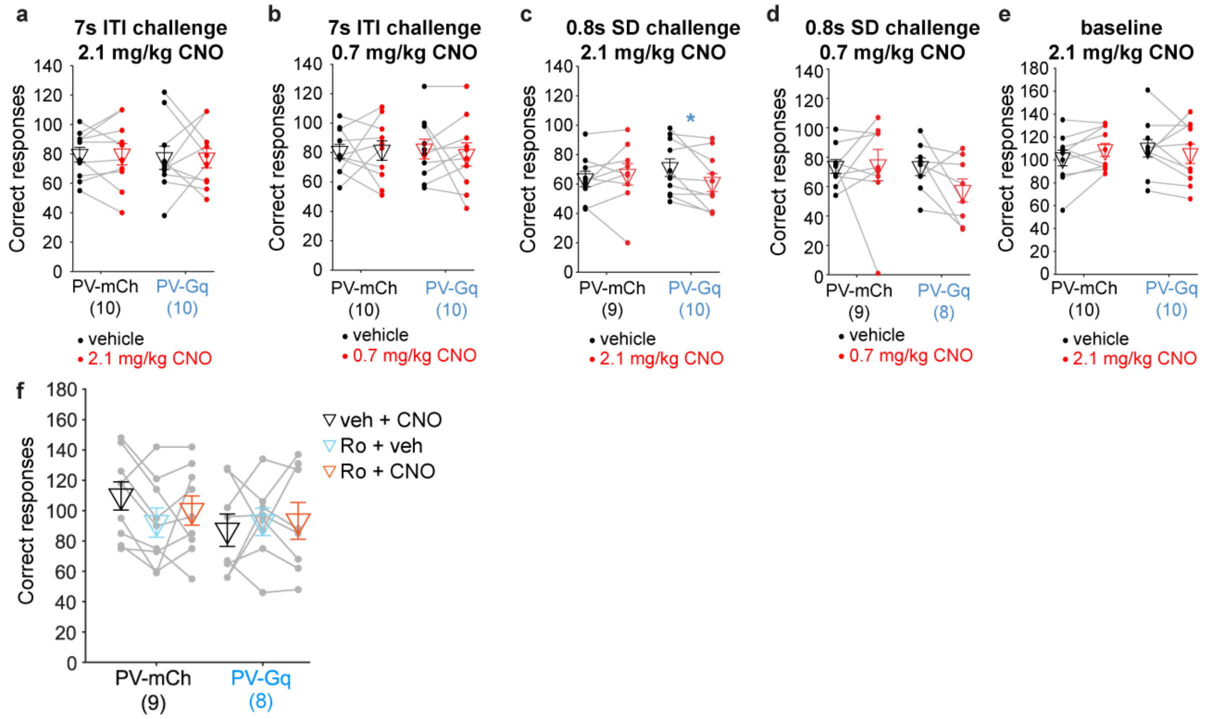

Supplementary Figure 4. Challenge-dependent changes of 5-CSRTT performance by PV-cell activation. (**a-e**) Total number of achieved correct responses in 7s-ITI challenge with 2.1 (a) or 0.7 (b) mg/kg CNO, the 0.8 s SD challenge with 2.1 (c) or 0.7 (d) mg/kg CNO, and the baseline condition with 2.1 mg/kg CNO (e). (**f**) Total number of achieved correct responses after application of vehicle and CNO (black), the impulsivity-inducing compound Ro63-1908 (Ro, 3 mg/kg) and vehicle (cyan), and combined pre-treatment with Ro and 0.7 mg/kg CNO (orange) in the baseline protocol in the groups stated on the x-axes (*N*-numbers in brackets; one animal per group was excluded because Ro alone did not induce >5 % premature responding). Drug effects were assessed within-subject with paired *t*-test. See Supplementary Table 4 for statistics. * $P < 0.05$. Non-significant pairwise comparisons ($P > 0.05$) not indicated. Error bars represent s.e.m. throughout.

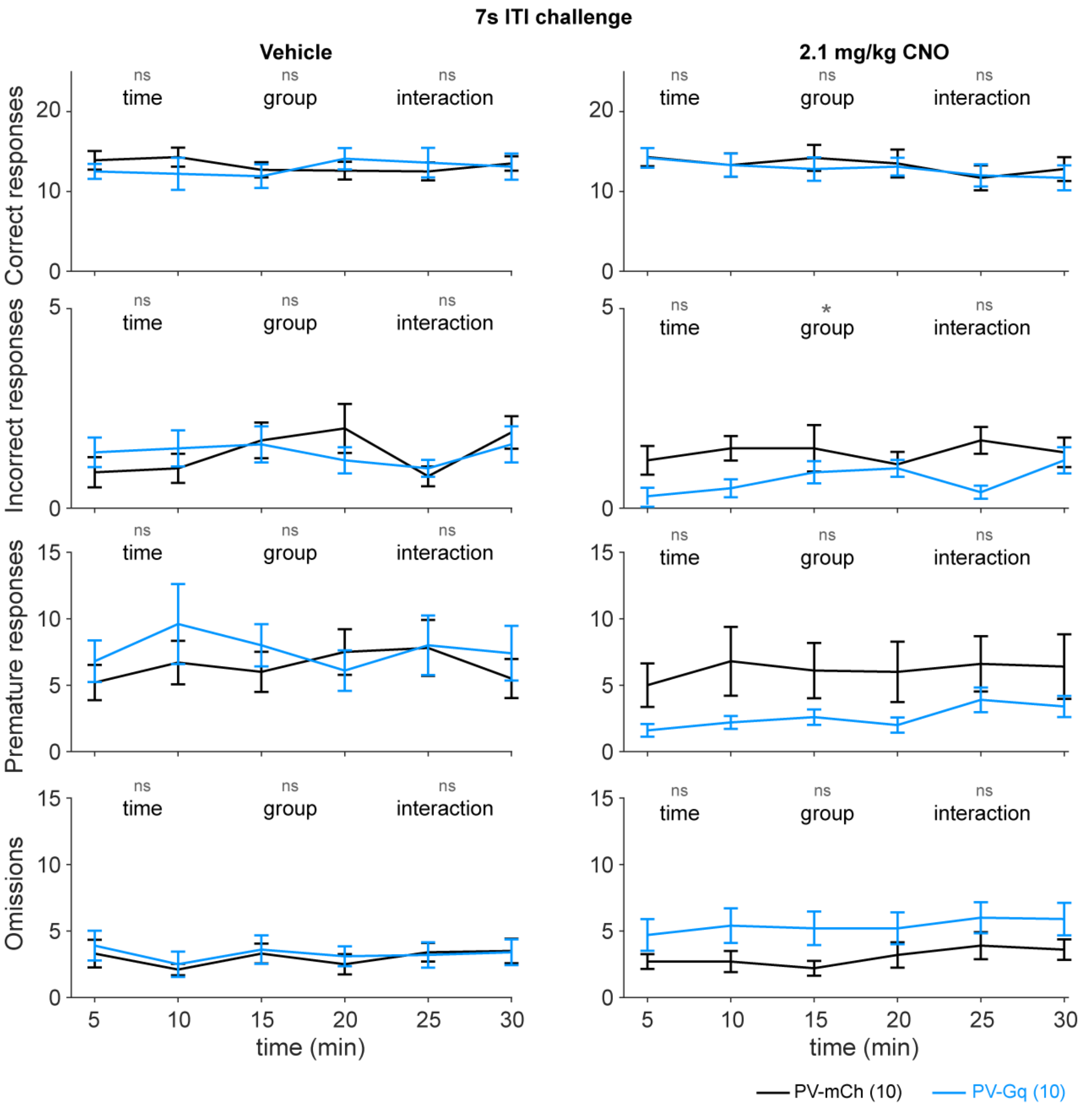

Supplementary Figure 5. Response numbers over time do not indicate adaptation or fatigue. (**a-b**) Total number of the responses of the type indicated on the left axes for the vehicle (left) and CNO (right) sessions analysed in 5 min-bins across the 30 min session and analysed with RM-ANOVA within each drug condition (7 s-ITI condition). No effects of time or time-group interactions are found for any condition indicating absence of within-session adaptation to the extended ITI challenge and fatigue. Differences between groups are found exclusively after CNO-application for incorrect responses. ^{ns} $P > 0.05$, * $P < 0.05$. Error bars represent s.e.m. throughout.

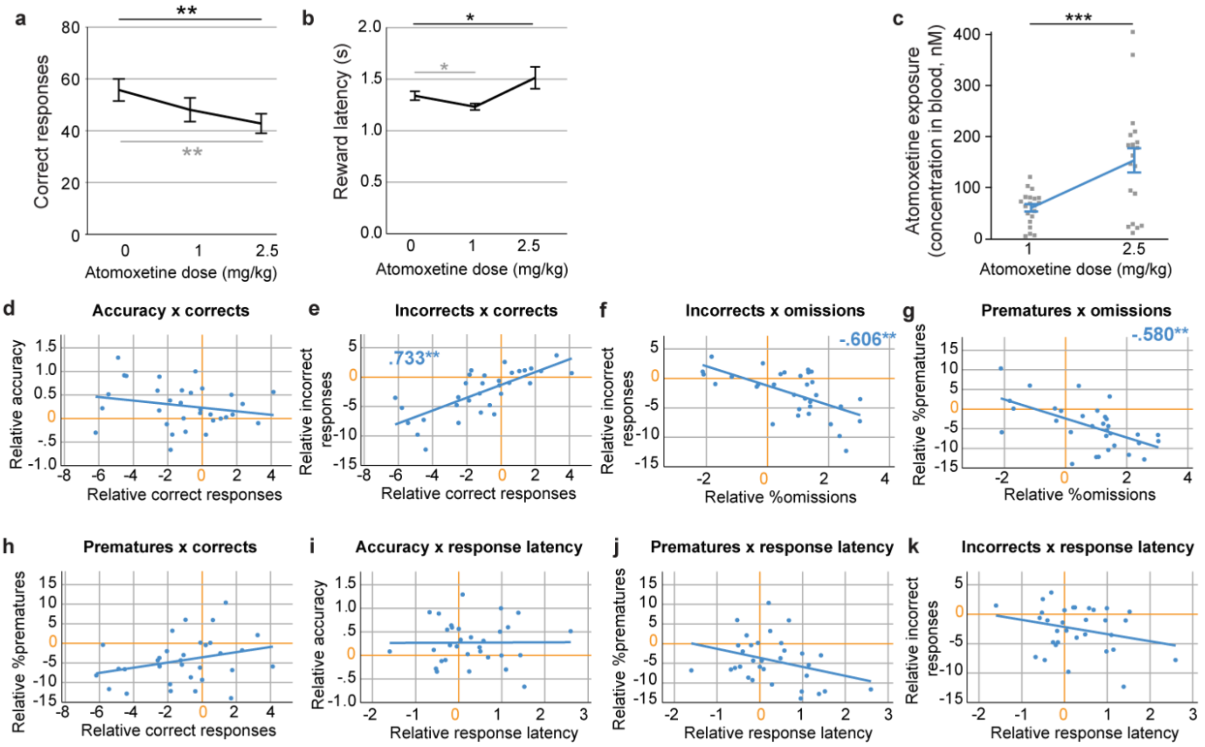

Supplementary Figure 6. Effects of atomoxetine on 5-CSRTT performance in C57BL/6J wildtype mice (*N* = 32). Extension of the analysis in main Figure 3. (**a-b**) Correct responses (a) and reward (b) latency after application of the stated doses of atomoxetine or vehicle in a challenge with 7s ITI and 0.8s SD. Results of within-subject RM-ANOVA (black) and paired Sidak post-hoc test between drug and vehicle conditions (grey) are indicated. (**c**) Concentration of atomoxetine in blood sampled 70-95 min after i.p. injection of the stated dose in a subset of animals (*N* = 20 each); asterisks refer to independent samples *t*-test. (**d-k**). Same bivariate correlation analysis as in main Figure 3r-u for the parameters stated above each panel; changes induced by 1 mg/kg atomoxetine relative to vehicle are plotted for each animal and fitted by a linear function. * $P < 0.05$, ** $P < 0.01$. Non-significant pairwise comparisons ($P > 0.05$) not indicated.

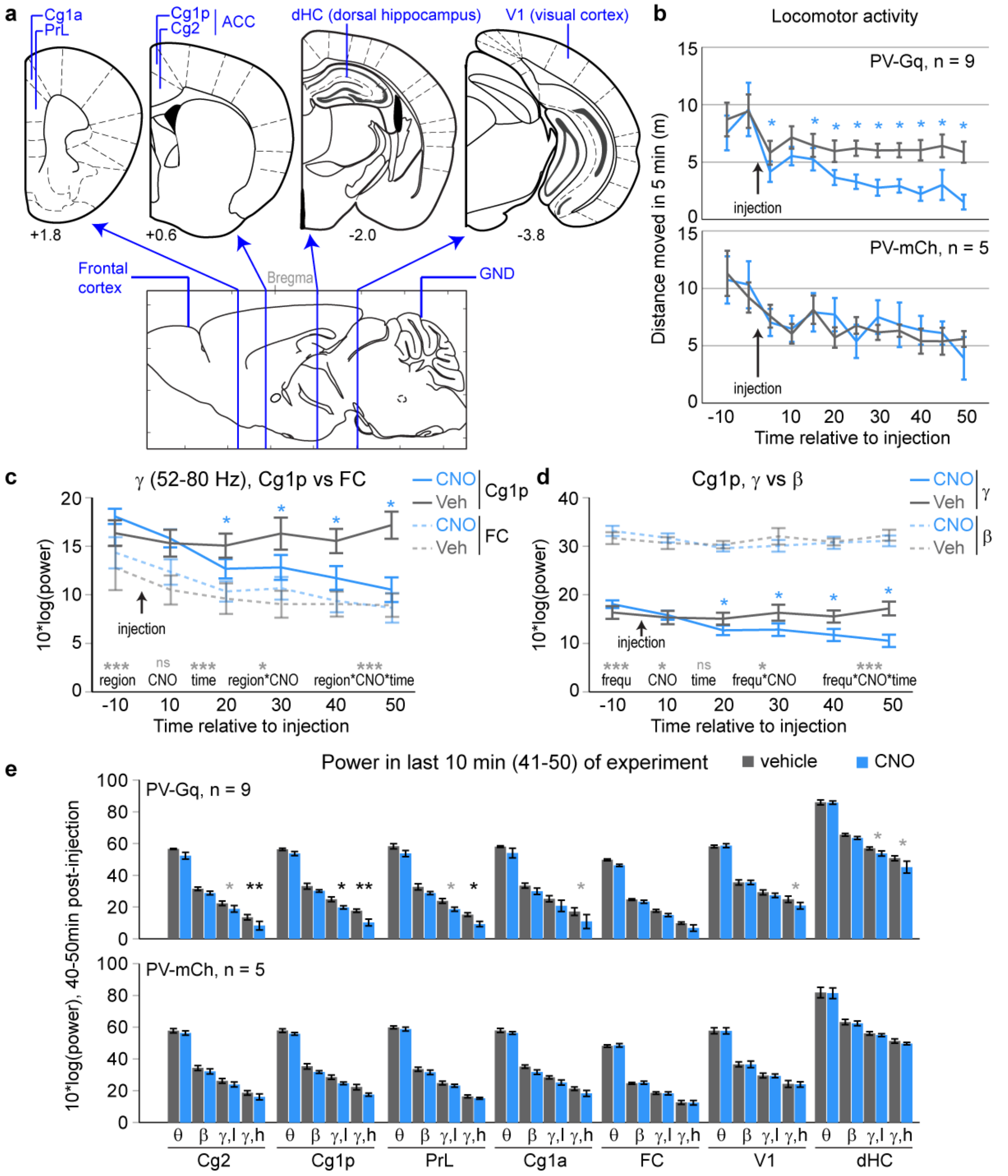

Supplementary Figure 7. PV-Gq activation in the ACC decreases predominantly high-gamma power of basal LFP oscillations in the open field. (**a**) Placement of implanted field electrodes illustrated in coronal (top) and saggital (bottom) projections. Drawings of brain slices according to the Franklin & Paxinos mouse brain atlas, 3rd ed.[12]; numbers in between indicate AP-coordinates of placements. (**b**) Distance moved in an open field from 10 min before until 50 min after injection of vehice or 2.1 mg/kg CNO (within-subject design) in simultaneously recorded PV-Gq (top) and PV-mCh (control, bottom) mice, shown in 5 min intervals. Within-subject Sidak post-hoc comparisons indicated after significant effect of CNO (RM-ANOVA). (**c-d**) Local power shown in 10 min bins from 10 min before until 50 min after

injection of vehice or 2.1 mg/kg CNO. Comparative within-subject analyses between high-gamma (52-80 Hz) power in transfected dorsal posterior ACC (Cg1p) and either high-gamma in FC (frontal cortex; c) or beta (15-30 Hz) in Cg1p (d) are illustrated with main effects of overall ANOVAs indicated at the bottom of each panel. The significant triple-interactions indicate that CNO induces a divergence selectively in the transfected cortical region (c) and gamma-frequency band (d) that develops over time, i.e. with increasing CNO bioavailability. Within-subject comparison are indicated where present, namely exclusively in gamma oscillations in Cg1p. (**e**) Analysis of the power in all analysed frequency bands and regions (indicated on x-axes) in PV-Gq (top) and PV-mCh (bottom) mice only for the last 10 min (minute 41-50 post-injection) of the test where the CNO-vehicle divergences appeared largest (see c, d); significant differences are mainly present in the high-gamma band in ACC but to a lesser degree also in connected regions PrL, dHC and V1; grey asterisks indicate significant differences that do not survive Bonferroni-correction for the number of frequency bands. Cg1/2, subregions of anterior (a) or posterior (p) ACC; dHC dorsal hippocampus; FC, frontal cortex; GND, ground electrode also used as reference; V1, primary visual cortex. θ, theta band (5-12 Hz); β, beta band (15-30 Hz); γ,l, low-gamma band (30-48 Hz); γ,h, high-gamma band (52-80 Hz). Vehicle, grey; CNO, blue. * $P < 0.05$, ** $P < 0.01$, *** $P < 0.001$; significance level not further specified for within-subject comparisons at individual time points in (b-d) for clarity. Non-significant pairwise comparisons ($P > 0.05$) not indicated. For ANOVAs, ^{ns} $P > 0.05$. Error bars, s.e.m.

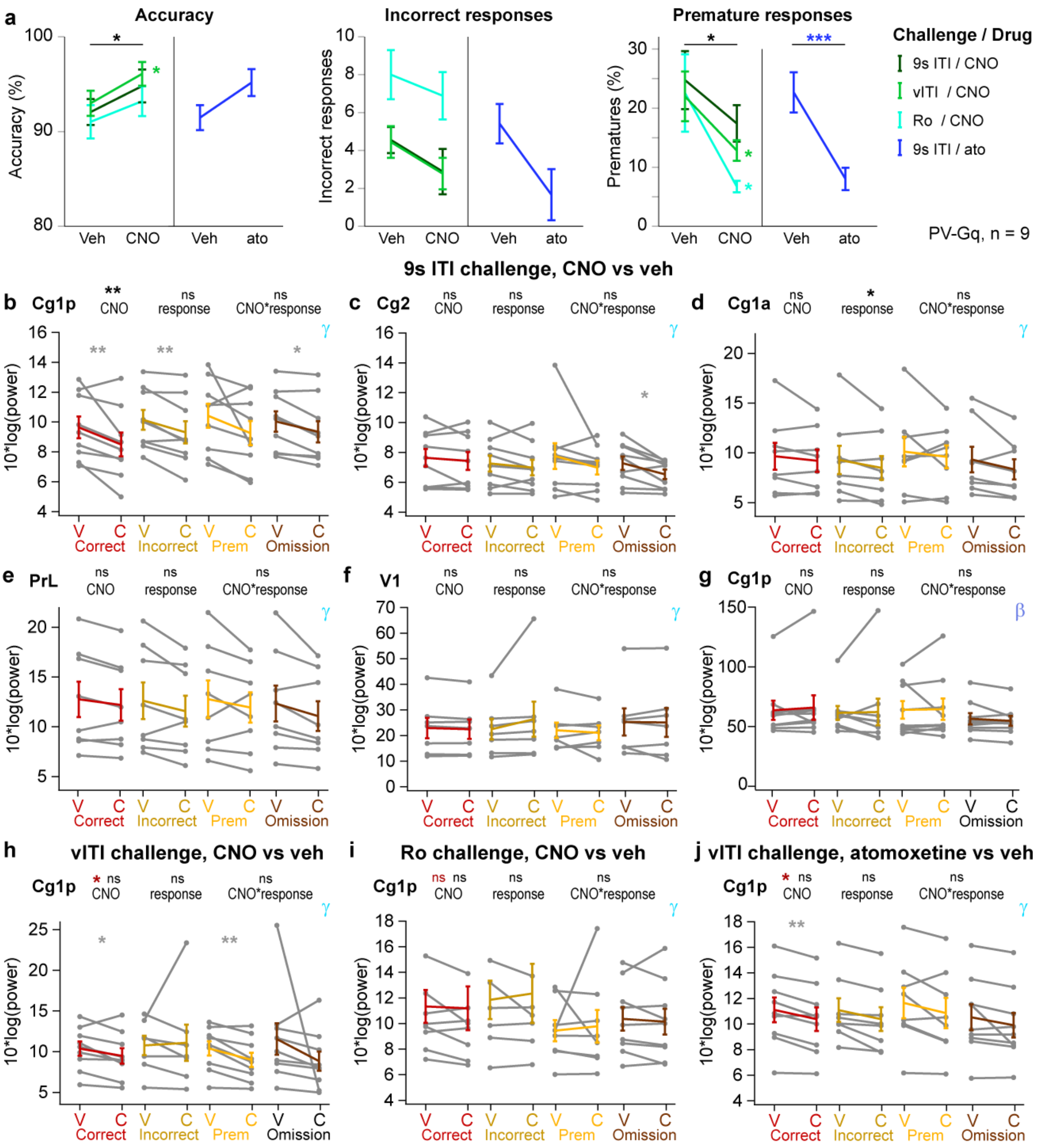

Supplementary Figure 8. PV-Gq activation in the ACC decreases high-gamma power of LFP oscillations during responses in the 5-CSRTT. (**a**) Behavioural performance of implanted and tethered PV-Gq mice performing the 5-CSRTT with simultaneous LFP-recordings and pre-treatment with either vehicle (Veh) or 2.1 mg/kg CNO (left) in three separate challenges (see colour-legend), or vehicle or 2.5 mg/kg atomoxetine (ato) in the 9s-ITI challenge. Results of overall RM-ANOVA across CNO doses and challenges (left) or paired *t*-tests (right) are indicated above each sub-panel; significant effects in individual chemogenetic experiments are indicated on the right side of each data line and colour-coded. The Ro-challenge corresponds to that applied in main Figure 2 (3 mg/kg Ro 63-1908 s.c.); the variable ITI (vITI) challenge involved a pseudo-random application of trials with an ITI of 7, 9, 11 and 13 s at equal probability. (**b-g**) Power of high-gamma (52-80 Hz; b-f) or beta (15-30 Hz;

g) oscillations under vehicle (V) or CNO (C) from 5 s before until 3 s after a given behavioural response as stated on x-axes; for omissions, the end of the SD was used as reference time point. Recording site stated in the top-left corner of each panel, frequency band in the top right corner. Results of RM-ANOVA across response types and drugs indicated at the top of each panel; results of paired Sidak post-hoc comparisons between drug and vehicle within each response type are indicated below in grey. (**h, i**) Same display and analysis as in (b) but for the vITI- (h) and the Ro-challenges (i). (**j**) Same display and analysis as in (b) but for the application of 2.5 mg/kg atomoxetine instead of CNO in the 9s-ITI challenge. * $P < 0.05$, ** $P < 0.01$, *** $P < 0.001$. Non-significant pairwise comparisons ($P > 0.05$) not indicated. For ANOVAs, ^{ns} $P > 0.05$. Red asterisks in (h-j) correspond to RM-ANOVAs calculated only with response types that occurred in every animal (some mice did not conduct any incorrect or premature response in those challenges leading to the loss of the subject across all response types for the ANOVA depicted by black symbols). Individual mice in grey, mean ± s.e.m. in colour.

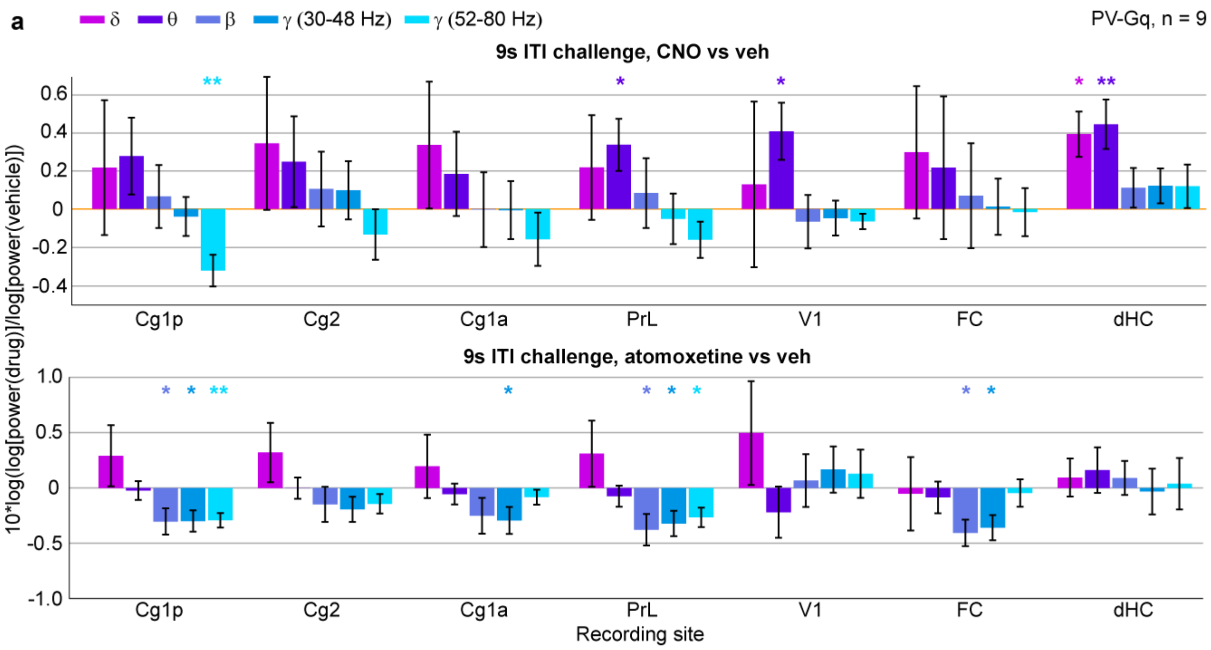

Supplementary Figure 9. PV-Gq activation in the ACC and atomoxetine both decrease high-gamma power of Cg1p LFP oscillations during correct responses in the 5-CSRTT. (**a**) Data from the 9s ITI challenge conducted with CNO (top) or atomoxetine treatment (bottom) as also shown in Supplementary Fig. 8, but only for data from correct responses. The power-value calculated in the colour-coded frequency band was retrieved for the vehicle and the drug conditions, and the log-transformed ratio [log($power_{drug}$)/log($power_{vehicle}$)] is plotted to allow for a comparison of regions and frequency bands (x-axes). Asterisks indicate results of one-sample *t*-tests against 0. * $P < 0.05$, ** $P < 0.01$. Non-significant pairwise comparisons ($P > 0.05$) not indicated. Note that PV-Gq activation and atomoxetine both decrease high-gamma power in the dorsal posterior ACC (Cg1p; see also Supplementary Fig. 8b, h, j).

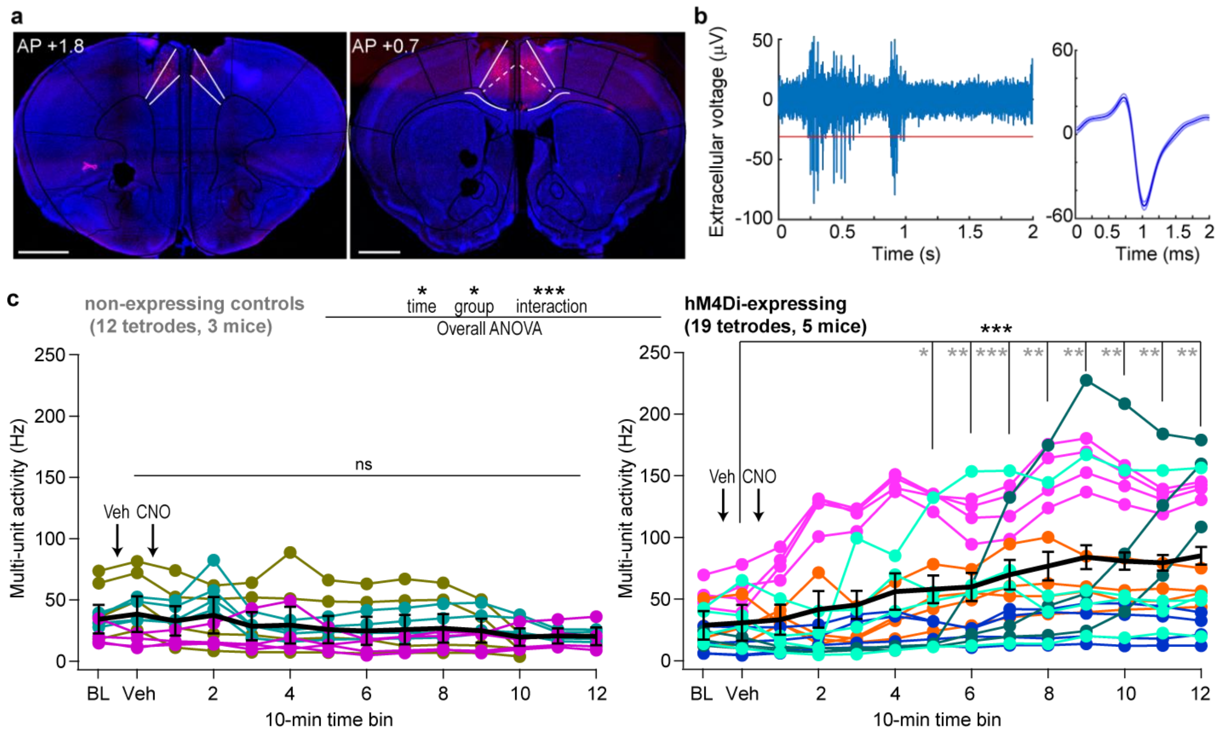

Supplementary Figure 10. Validation of effect of hM4Di activation in ACC PV-interneurons. (**a**) Transfection of PV-interneurons with hM4Di-mCherry (red); ACC is indicated by white borders hand-drawn according to slice layout and Franklin & Paxinos mouse brain atlas, 3rd ed.[12]. (**b**) Left: Example of multi-unit activity (MUA) recording of 2 s in ACC with action potentials extracted by threshold criterion (threshold indicated by red line). Right: Average waveform of extracted action potentials from the same recording, with shaded envelope indicating the standard deviation. (**c**) MUA activity in ACC of untransfected PV-Cre controls (left; 12 tetrodes from 3 mice) or hM4Di-expressing PV-Cre mice (right; 19 tetrodes from 5 mice) shown for a 10 min baseline period (BL), a subsequent 10 min episode after injection of vehicle (Veh), and 12 further 10 min time bins that followed the subsequent injection of 10 mg/kg CNO. Individual tetrodes are shown in shades of grey or green; within-group average shown in black. Results of overall RM-ANOVA using the Veh time bin in addition to all subsequent CNO time bins are shown at the top, indicating a significant time-group interaction. Paired Sidak post-hoc comparisons between the Veh time bin and each CNO time bin indicated a higher MUA from ca. 30-40 min onwards and for the remaining recording time (grey indicators) in hM4Di-expressing mice, but not in control mice, suggesting a CNO/hM4Di-induced disinhibitory effect on the ACC circuit as expected from an inhibition of inhibitory interneurons. As one control mice was recorded for 100, instead of 120 min post-injection, indicators reflect an ANOVA across all mice until 100 min after CNO, while another RM-ANOVA was calculated for the reduced number of mice for the last two time points; overall significance levels were identical between both calculations. * $P < 0.05$, ** $P < 0.01$, *** $P <$

0.001. Non-significant pairwise comparisons ($P > 0.05$) not indicated. For ANOVAs, ^{ns} $P > 0.05$.

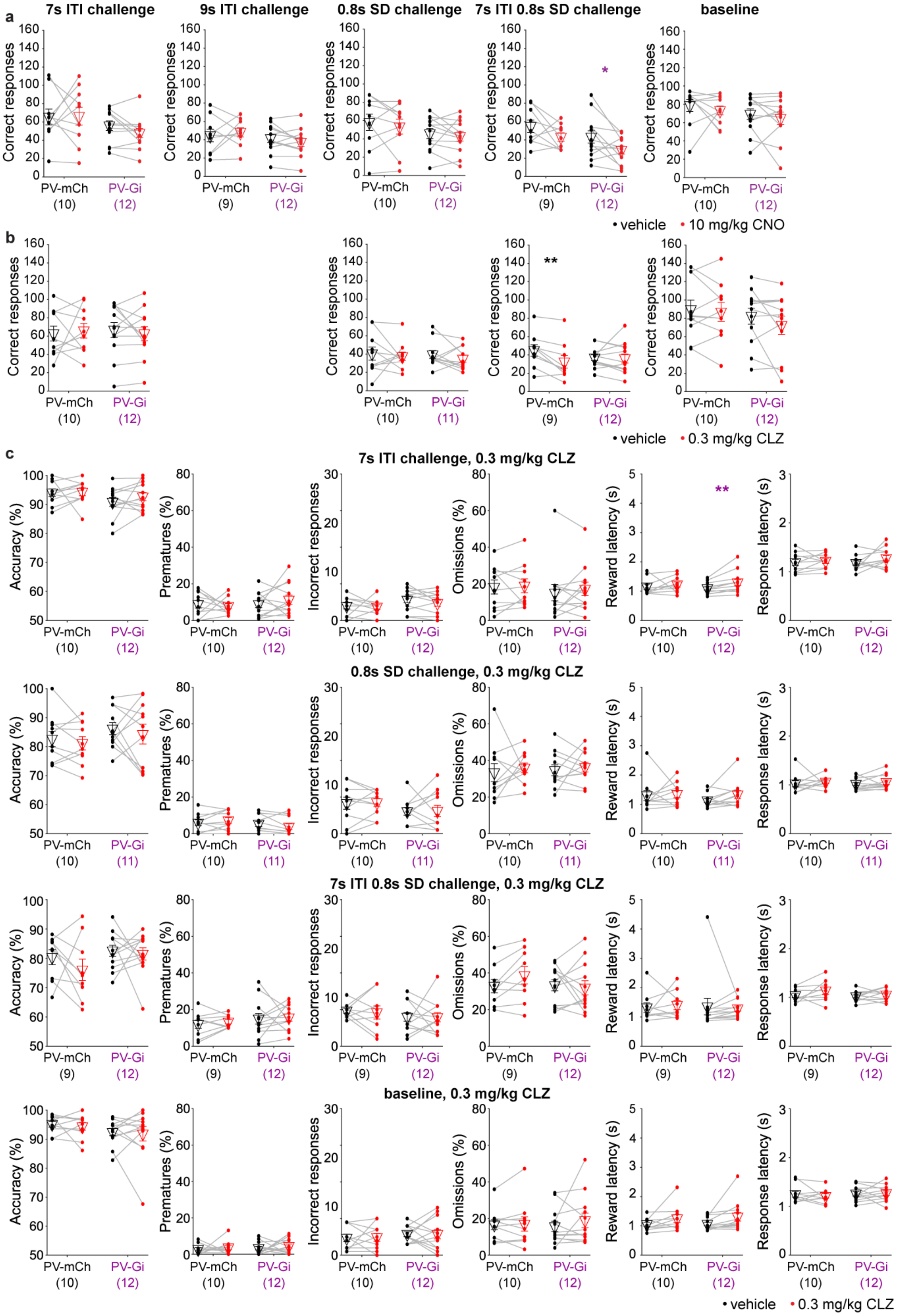
a
7s ITI challenge
9s ITI challenge
0.8s SD challenge
7s ITI 0.8s SD challenge
baseline
Correct responses
PV-mCh (10)
PV-Gi (12)
PV-mCh (9)
PV-Gi (12)
*
• vehicle • 10 mg/kg CNO
b
Correct responses
PV-mCh (10)
PV-Gi (12)
PV-Gi (11)
PV-mCh (9)
**
• vehicle • 0.3 mg/kg CLZ
c
7s ITI challenge, 0.3 mg/kg CLZ
Accuracy (%)
Prematures (%)
Incorrect responses
Omissions (%)
Reward latency (s)
Response latency (s)
**
0.8s SD challenge, 0.3 mg/kg CLZ
7s ITI 0.8s SD challenge, 0.3 mg/kg CLZ
baseline, 0.3 mg/kg CLZ
PV-mCh (10)
PV-Gi (11)
PV-mCh (9)
PV-Gi (12)
• vehicle • 0.3 mg/kg CLZ

Supplementary Figure 11. Decreased number of correct responses due to PV-cell inhibition in combined challenge. (**a-b**) Number of correct responses for the groups stated on the x-axis in the challenges stated above panel (a) conducted either with 10 mg/kg CNO (a) or 0.3 mg/kg CLZ in the PV-Gi group. (**c**) Same display and analysis as in main Fig. 4, but for the experiments conducted 0.3 mg/kg CLZ as DREADD agonist; in the following order: baseline, 7s-ITI challenge, 0.8s-SD challenge, combined 7s-ITI/0.8s-SD challenge. Asterisks indicate within-subject comparisons (paired *t*-test). See Supplementary Table 5 for further statistics with RM-ANOVA and post-hoc tests. * $P < 0.05$. Non-significant pairwise comparisons ($P > 0.05$) not indicated. Error bars represent s.e.m. throughout.

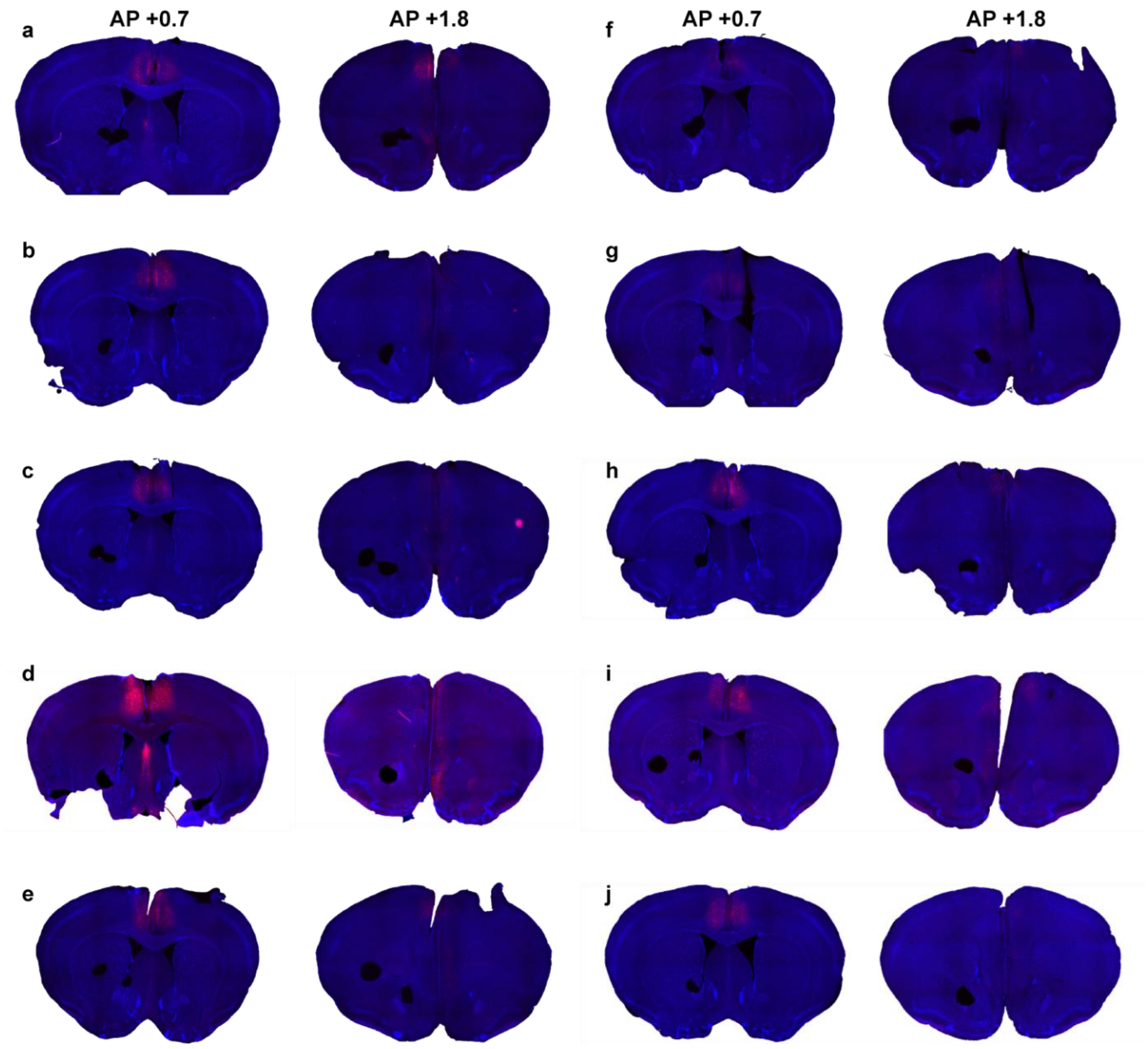

Supplementary Figure 12. Targeting of ACC Sst-interneurons with hM3Dq across animals. (**a-j**) hM3Dq-mCherry expression in every mouse (a-j) of the PV-Gq cohort at the approximate anterior-posterior (AP) sites of transfection.

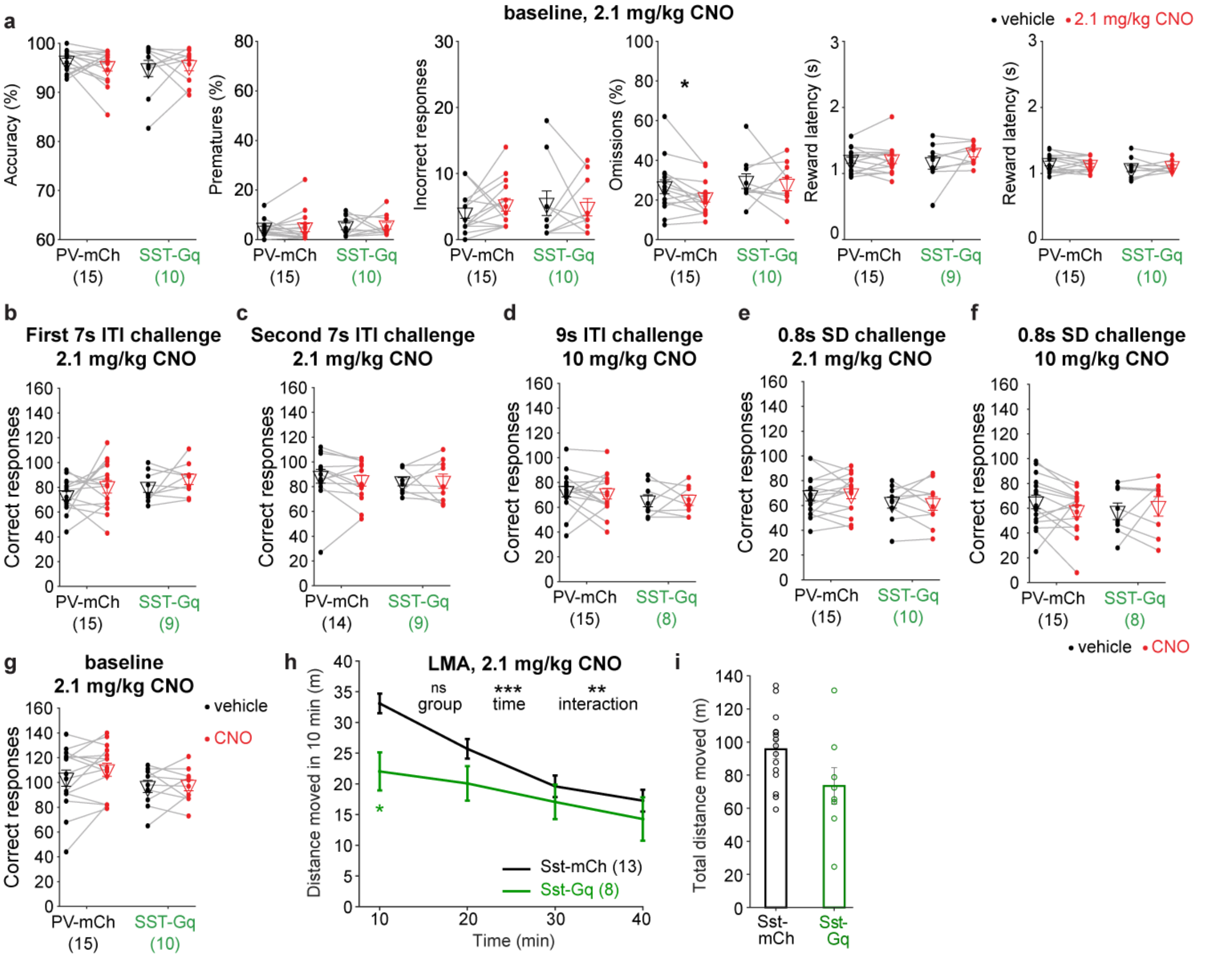

Supplementary Figure 13. Limited effect of activation of ACC Sst-interneurons on impulsivity and attention. (**a**) From left to right (as indicated on y-axes): Attentional accuracy, premature and incorrect responding, omissions and reward and response latencies in the 5-CSRTT baseline protocol; plotted for each individual mouse of the groups stated on the x-axes (*N*-numbers in brackets) for pretreatment with CNO (red dots) or vehicle (black dots). (**b-g**) The number of correct responses shown for all challenges conducted in the Sst-Gq cohort, as named above each panel; otherwise same display as in panel (a) and main Fig. 5. Green and black symbols above data lines indicate significant within-subject differences between vehicle and CNO pretreatment (paired *t*-test). See Supplementary Tables 6-7 for further statistics with RM-ANOVA and post-hoc tests, and reasons for varying *N*-numbers. In some cases, average reward latency values of individual mice had to be excluded from the datasets because they were unrealistically low (< 0.3 s) or high (> 10 s) due to a technical error of detecting receptacle entries (break-beams getting occluded by milk-reward). (**h**) Locomotor activity (LMA) after injection of 2.1 mg/kg CNO displayed in 10 min-intervals and analysed with RM-ANOVA (main effects stated at top of panel; between-subject design), and pairwise Sidak post-hoc test (green asterisk). (**i**) Total distance moved in LMA test; *P*-value indicates *t*-test. *

$P < 0.05$; ** $P < 0.01$, *** $P < 0.001$. Non-significant pairwise comparisons ($P > 0.05$) not indicated. For ANOVAs, ^{ns} $P > 0.05$. Error bars represent s.e.m. throughout.

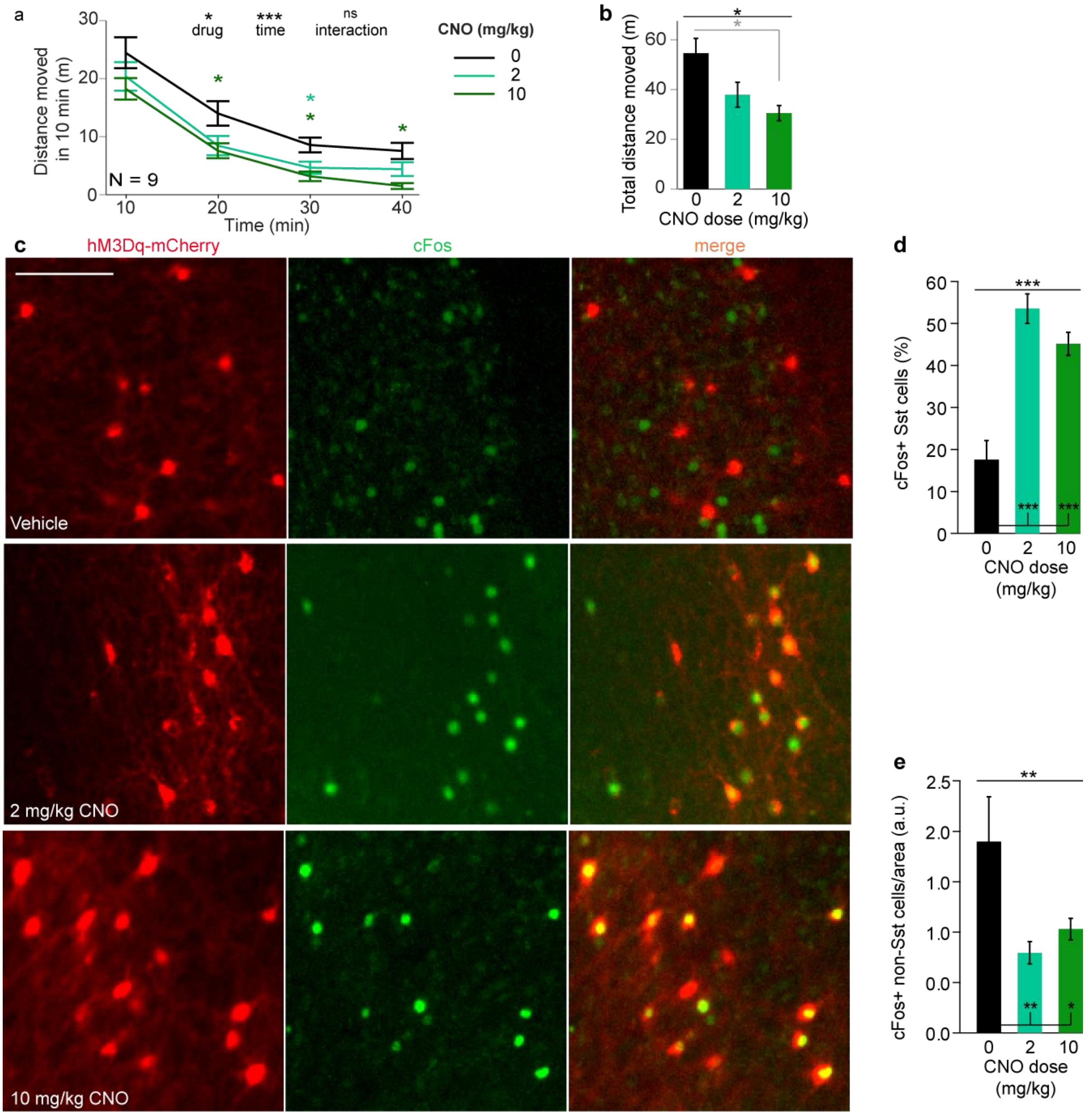

Supplementary Figure 14. Validation of hM3Dq-mediated activation of ACC Sst-interneurons. (**a**) Distance moved in a novel open field 15 min after application of vehicle, 2 or 10 mg/kg CNO, calculated in 10 min intervals. Results of overall RM-ANOVA across doses and time bins are shown at the top of the panel, pairwise differences (Sidak-adjusted) are indicated directly above the data coded by the colour of the respective CNO dose. Within-subject design. (**b**) Same data as in (a) but plotted as total distance moved and analysed by RM-ANOVA across doses and subsequent Sidak post-hoc test only. (**c**) Example fluorescence microscopy images from the ACC region of brain slices from Sst-Cre mice pre-treated with vehicle, 2 or 10 mg/kg CNO (as indicated) in their home-cage ca. 90 min before perfusion, stained against cFos (green, middle); native hM3Dq-mCherry expression in red (left); merged images on the right. Note the cFos-expression in mCherry-positive (Sst+) after CNO-treatment, while cFos expression outside red cell bodies decreases. (**d-e**) Quantification of results of histological experiments by ratio of cFos-positive among mCherry-positive cells (d) and density of mCherry-negative cFos-positive cells per unit area (e). Results have been obtained from 3 mice per dose and the following number of slices have been analysed: vehicle,

26 (d) and 8 (e); 2 mg/kg CNO, 28 (d) and 10 (e); 10 mg/kg CNO, 25 (d) and 12 (e). Results of one-way ANOVA (top) and pairwise Sidak post-hoc comparison with vehicle group (bottom) are shown. * $P < 0.05$; ** $P < 0.01$, *** $P < 0.001$. Non-significant pairwise comparisons ($P > 0.05$) not indicated. For ANOVAs, ^{ns} $P > 0.05$. Error bars, s.e.m.

Supplementary Tables

Supplementary Table 1. Training and test stages of the 5-CSRTT.

a

	**Parameters of training**			**Criteria for stage transition (to be met on 1 day)**			
**Stage**	SD (s)	LH (s)	ITI (s)	# correct	% correct	% accuracy	%omissions
**1**	20	22	2	>= 30	>= 40	-	-
**2**	8	10	2	>= 40	>= 50	-	-
**3**	8	10	5			>= 80	<= 50
**4**	4	6	5			>= 80	<= 50
**5, BL**	2	4	5			>= 80	<= 50
**Challenge**							
6	0.8	2.8	5	0.8 SD (attention) challenge,			
7	2	4	7	7s fixed ITI challenge			
8	2	4	9	9s fixed ITI challenge			
9	0.8	2.8	7	Combined SD/ITI challenge			

b

**PV-Gq (behaviour only)**			
Challenge	Dose (mg/kg)	Days within experiment	Weeks after same challenge
7s ITI	2.1	7 / 8 *	na
0.8s SD	2.1	7	na
Baseline	2.1	7	na
7s ITI	0.7	7	24 / 13 *
0.8s SD	0.7	3	36 / 20 *
Ro63-1908	0.7	7 (2x)	1 **

**PV-Gq (electrophysiology)**				
Challenge	Drug	Dose (mg/kg)	Days within experiment	Weeks after same challenge
9s ITI	CNO	2.1	7	na
vITI SD	CNO	2.1	5	na
Ro63-1908	CNO	2.1	7	na
9s ITI	Ato	2.5	6	4

**Sst-Gq**			
Challenge	Dose (mg/kg)	Days within experiment	Weeks after same challenge
7s ITI	2.1	7	na
0.8s SD	2.1	7	na
Baseline	2.1	7	na
7s ITI (2nd)	2.1	7	13 / 5 *
9s ITI	10	7	na ***
0.8s SD	10	6 / 4 *	16 / 8 *

**PV-Gi**				
Challenge	Drug	Dose (mg/kg)	Days within experiment	Weeks after same challenge
7s ITI	CNO	10	3	na
0.8s SD	CNO	10	3	na
Baseline	CNO	10	5	na
9s ITI	CNO	10	7	na
0.8s SD / 7s ITI	CNO	10	5	10
7s ITI	CLZ	0.3	3	8
0.8s SD	CLZ	0.3	3	8
Baseline	CLZ	0.3	3	na
0.8s SD / 7s ITI	CLZ	0.3	7	na

**C57bl/6 wildtype cohort (atomoxetine application)**			
Challenge	Dose (mg/kg)	Days within experiment	Weeks after same challenge
0.8s SD / 7s ITI	1.0; 2.5	6-7 (2x)	4

(**a**) Parameters of the training and test stages. The limited hold (LH) period includes the SD. **BL**, baseline stage which was used for continuous re-training in between challenge experiments and also for chemogenetic testing (baseline protocol only) itself. (**b**) Lists of the applied challenges in the five cohorts described in this manuscript of which the corresponding main data is shown in Fig. 1-3 [PV-Gq (behaviour only)], Fig. 3 (C57bl/6 wildtype cohort), Fig. 4 (PV-Gi), Fig. 5 (Sst-Gq), and Supplementary Fig. 7-9 [PV-Gq (electrophysiology)], stating the dose of the applied drug (CNO, CLZ or atomoxetine; Ro63-1908 dose of 3 mg/kg not separately stated), the number of days between the repetition of the challenge condition for within-subject comparisons between doses and, where applicable, the number of weeks

between the repeated application of the same challenge condition for another experiment. Note that, in the PV-Gq (behaviour) and Sst-Gq cohorts, some further chemogenetic 5-CSRTT experiments have been tried towards the end of the experimental schedule, mostly only in a subset of animals, but have not been further pursued due to technical failures or redundancy with previous experiments. Also, in the PV-Gq(electrophysiology) cohort, two challenges without chemogenetic modulation were conducted before the series of chemogenetic experiments started. Remarks: * Two values are stated referring to two parts of the cohort that were run separately due to logistic limitations. ** A Ro-challenge was conducted already 7d before the actual 3-session sequence of this experiment to avoid increases in variance between the first and second Ro-application of the actual experiment. *** Note, that this 9s-ITI challenge was conducted either 1 or 2 wks after the last previous 7s-ITI challenge, which is why the ITI was increased.

Supplementary Table 2. Statistical assessment of CNO-induced 5-CSRTT behaviour in the PV-Gq cohort.

Chemogenetic experiments in PV-Gq cohort

Protocol, CNO dose		Behavioural parameter	Figure	RM ANOVA							Sidak post-hoc tests: p-value			
					dose		group		interaction		Gq vs Ctrl		Vehicle vs CNO	
				df1,df2	F	p	F	p	F	p	Vehicle	CNO	Gq	Ctrl
**7 s ITI**		%premature	**1e**	1, 18	9.09	0.0074	0.46	0.5083	5.79	0.0271	0.5950	0.0890	0.0012	0.6720
2.1 mg/kg		accuracy	**1e**	1, 18	2.28	0.1482	0.92	0.3510	4.47	0.0487	0.7056	0.0442	0.0195	0.6744
**Group**	**N**	%omissions	**1e**	1, 18	3.59	0.0742	2.19	0.1563	3.55	0.0757	0.8095	0.0542	0.0155	0.9941
Ctrl	10	#correct	**S4**	1, 18	0.00	1.0000	0.09	0.7734	0.00	0.9518				
Gq	10	#incorrect	**1e**	1, 18	3.18	0.0916	1.79	0.1979	3.51	0.0773	1.0000	0.0191	0.0187	0.9492
		%perseverative	-	1, 18	0.06	0.8070	0.21	0.6550	0.53	0.4756				
		reward latency	**1e**	1, 18	0.09	0.7730	0.01	0.9438	0.00	0.9680				
		response latency	**1e**	1, 18	0.02	0.9036	0.99	0.3340	0.48	0.4985				
		CV(response latency)	-	1, 18	0.01	0.9363	1.06	0.3175	1.54	0.2300				
**0.8 s SD**		%premature	**1g**	1, 18	0.19	0.6641	1.49	0.2376	0.00	0.9883				
2.1 mg/kg		accuracy	**1g**	1, 18	0.72	0.4081	1.90	0.1847	1.01	0.3276				
**Group**	**N**	%omissions	**1g**	1, 18	8.32	0.0099	0.65	0.4319	2.71	0.1173	0.8814	0.1939	0.0049	0.3927
Ctrl	9	#correct	**S4**	1, 18	1.40	0.2526	0.09	0.7735	5.42	0.0317	0.2594	0.6304	0.0231	0.4279
Gq	10	#incorrect	**1g**	1, 18	2.97	0.1020	4.10	0.0579	1.07	0.3149	0.0737	0.2980		
		%perseverative	-	1, 18	0.80	0.3822	0.27	0.6117	0.07	0.7963				
		reward latency	**1g**	1, 18	0.45	0.5123	0.88	0.3601	3.17	0.0921	0.9455	0.1721	0.1006	0.4424
		response latency	**1g**	1, 18	0.17	0.6885	2.29	0.1473	0.07	0.7882				
		CV(response latency)	-	1, 18	0.66	0.4255	0.22	0.6460	3.93	0.0630	0.1311	0.4503	0.4202	0.0634
**Baseline**		%premature	**1i**	1, 18	2.65	0.1208	0.61	0.4455	4.60	0.0459	0.9935	0.1207	0.0157	0.7194
2.1 mg/kg		accuracy	**1i**	1, 18	3.17	0.0921	0.35	0.5619	0.06	0.8105			0.2918	0.1698
**Group**	**N**	%omissions	**1i**	1, 18	0.00	0.9920	0.14	0.7151	1.59	0.2228				
Ctrl	10	#correct	**S4**	1, 18	0.22	0.6411	0.03	0.8755	1.68	0.2108				
Gq	10	#incorrect	**1i**	1, 18	4.21	0.0550	0.67	0.4247	0.00	0.9695			0.1566	0.1717
		%perseverative	-	1, 18	0.01	0.9228	0.02	0.9002	0.15	0.7029				
		reward latency	**1i**	1, 18	0.49	0.4921	0.14	0.7117	0.07	0.7973				
		response latency	**1i**	1, 18	4.59	0.0461	0.33	0.5752	0.24	0.6305			0.0791	0.2576
		CV(response latency)	-	1, 18	4.63	0.0452	0.36	0.5565	0.12	0.7310			0.2184	0.0938
**7 s ITI**		%premature	**1f**	1, 18	0.47	0.5006	3.54	0.0760	1.03	0.3231	0.4836	0.0783		
0.7 mg/kg		accuracy	**1f**	1, 18	3.43	0.0805	4.83	0.0412	2.75	0.1148	0.4791	0.0155	0.0232	0.8920
**Group**	**N**	%omissions	**1f**	1, 18	2.34	0.1438	3.32	0.0849	3.96	0.0621	0.4447	0.0360	0.0229	0.7485
Ctrl	10	#correct	**S4**	1, 18	0.07	0.7928	0.01	0.9432	0.16	0.6972				
Gq	10	#incorrect	**1f**	1, 18	5.59	0.0295	4.90	0.0401	2.89	0.1065	0.4002	0.0091	0.0101	0.6438
		%perseverative	-	1, 18	0.07	0.7990	2.69	0.1186	0.25	0.6253				
		reward latency	**1f**	1, 18	2.35	0.1440	2.92	0.1056	0.32	0.5802				
		response latency	**1f**	1, 18	0.27	0.6113	3.10	0.0953	5.21	0.0349	0.7258	0.0122	0.0633	0.2281
		CV(response latency)	-	1, 18	0.00	0.9905	0.25	0.6230	0.00	0.9675				
**0.8 s SD**		%premature	**1h**	1, 15	2.49	0.1351	0.66	0.4277	0.89	0.3594				
0.7 mg/kg		accuracy	**1h**	1, 15	0.03	0.8610	0.16	0.6977	1.63	0.2217				
**Group**	**N**	%omissions	**1h**	1, 15	1.86	0.1932	3.45	0.0829	4.90	0.0427	0.7108	0.0128	0.0266	0.5438
Ctrl	9	#correct	**S4**	1, 15	0.45	0.5109	3.50	0.0811	7.03	0.0182	0.7108	0.0128	0.0373	0.1700
Gq	8	#incorrect	**1h**	1, 15	0.49	0.4938	0.12	0.7359	0.80	0.3856				
		%perseverative	-	1, 15	0.00	0.9810	1.94	0.1837	1.40	0.2544				
		reward latency	**1h**	1, 15	0.90	0.3570	3.37	0.0862	0.53	0.4769	0.1848	0.0267		
		response latency	**1h**	1, 15	1.98	0.1796	3.17	0.0955	1.57	0.2289	0.3349	0.0439		
		CV(response latency)	-	1, 15	0.00	0.9646	0.50	0.4904	1.20	0.2909				

Results of repeated-measures ANOVA (left), pairwise between-subject and paired within-subject Sidak-adjusted simple-main effects post-hoc tests (middle) for the experiments shown in Figure 1 and Supplementary Fig. 4 conducted in PV-Cre mice transfected with hM3q or mCherry. All repeated-measures ANOVAs are two-way ANOVAs involving one within-subject parameter (CNO or drug) and one between-subject parameter (subgroup, identified in the small tables on the left with contributing N-numbers. *Reasons for varying N-numbers:* One mCh-Control was excluded from the 0.8s-SD challenge with 2.1 mg/kg CNO because the wrong compound was injected; one mCh-Control and one Gq mouse were excluded from the

0.8s-SD challenge with 0.7 mg/kg CNO because they did not participate in the task (1-2 number corrects) and from one further Gq-mouse data was lost due to a technical error. The analysed challenge protocol and applied drug are identified at the top-left of each section. The figure that displays the statistically tested data is shown in the figure panel indicated in the "Figure" column. For the parametric challenges, the degrees of freedom (df1, df2) are identical for all three factors of the ANOVA and hence only stated once. Post-hoc tests that were not indicated to be run because of a lack of significant between-subject, within-subject or interaction effects in the overall RM-ANOVA are omitted (grey cells). *P*-values < 0.01 are highlighted in dark green, *P*-values < 0.05 in light green, *P*-values < 0.1 in light grey-green.

Supplementary Table 3. Statistical assessment of CNO-induced 5-CSRTT behaviour in the PV-Gq cohort, analysis across multiple challenges.

RM ANOVA: combined analysis of 0.7 and 2.1mg/kg CNO in 7s-ITI challenges; Gq-mice only

PV-Gq: n = 10		dose		drug		dose*drug	
	df1,df2	F	p	F	p	F	p
%premature	1, 9	8.06	0.0194	12.92	0.0058	5.10	0.0503
accuracy	1, 9	4.45	0.0642	20.27	0.0015	0.13	0.7229
%omissions	1, 9	0.87	0.3751	8.08	0.0193	0.01	0.9365
#incorrect	1, 9	3.39	0.0986	22.28	0.0011	0.58	0.4654

RM ANOVA: combined analysis of 7s-ITI and 0.8s-SD challenges, 2.1mg/kg CNO; Gq-mice only

PV-Gq: n = 10		challenge		drug		challenge*drug	
	df1,df2	F	p	F	p	F	p
%premature	1, 9	17.53	0.0024	6.45	0.0318	8.44	0.0174
accuracy	1, 9	10.32	0.0106	1.51	0.2496	1.83	0.2088
%omissions	1, 9	101.57	0.0000	14.61	0.0041	0.16	0.6969
#incorrect	1, 9	5.16	0.0492	3.01	0.1170	0.91	0.3645

RM ANOVA: combined analysis of 7s ITI and 0.8s SD challenges, 0.7mg/kg CNO; Gq-mice only

PV-Gq: n = 8		challenge		drug		challenge*drug	
	df1,df2	F	p	F	p	F	p
%premature	1, 7	0.57	0.4739	5.64	0.0493	1.29	0.2932
accuracy	1, 7	7.90	0.0261	1.19	0.3120	5.32	0.0545
%omissions	1, 7	8.29	0.0237	21.25	0.0025	0.00	0.9986
#incorrect	1, 7	6.54	0.0377	2.72	0.1432	3.66	0.0973

Results of 2-way repeated-measures ANOVA within the subgroups of Gq-transfected PV-Cre mice involving data from two separate experiments, representing distinct dose-levels of CNO or distinct challenge-protocols, for the experiments shown in Figure 1 and Supplementary Fig. 4 and identified at the top if each sub-table. All repeated-measures ANOVAs are two-way ANOVAs involving two within-subject parameters [drug (CNO vs. vehicle), and either dose (0.7 vs. 2.1 mg/kg CNO) or challenge (7s-ITI or 0.8s-SD)]. Reasons for varying *N*-numbers are as stated in the legend of Supplementary Table *2.* The degrees of freedom (df1, df2) are identical for all factors of the ANOVA and hence only stated once. *P*-values < 0.01 are highlighted in dark green, *P*-values < 0.05 in light green, *P*-values < 0.1 in light grey-green.

Supplementary Table 4. Statistical assessment of CNO-induced 5-CSRTT behaviour in the PV-Gq cohort in the Ro-challenge.

3 mg/kg Ro, 0.7mg/kg CNO		RM ANOVA									Univariate tests, p		Sidak post-hoc tests: p-value				
Behavioural parameter	Figure	dose			group			interaction			Effect of dose		Gq vs Ctrl @condition			V/Ro v CNO_Ro	
		df1,df2	F	p	df1,df2	F	p	df1,df2	F	p	Gq	Ctrl	V/Ro	CNO/V	CNO/Ro	Gq	Ctrl
%premature	**2b**	2, 30	20.20	0.0000	1, 15	6.29	0.0241	2, 30	3.46	0.0446	0.0090	0.0001	0.3686	0.2742	0.0082	0.0708	0.9949
accuracy	**2a**	2, 30	14.98	0.0000	1, 15	2.50	0.1345	2, 30	1.90	0.1667	0.0501	0.0014				0.1115	0.6204
%omissions	**2d**	2, 30	16.95	0.0000	1, 15	10.94	0.0048	2, 30	4.36	0.0217	0.0020	0.0038	0.1219	0.0236	0.0010	0.0018	0.8486
#correct	**S4**	2, 30	0.52	0.6011	1, 15	0.61	0.4483	2, 30	1.86	0.1731							
#incorrect	**2c**	2, 30	15.26	0.0000	1, 15	3.34	0.0877	2, 30	1.64	0.2117	0.0179	0.0040	0.2969	0.2251	0.0440	0.0645	0.8902
reward latency	**2e**	2, 30	61.79	0.0000	1, 15	1.63	0.2243	2, 30	0.24	0.7865	0.0001	0.0000				0.3877	0.8061
response latency	**2f**	2, 30	6.13	0.0059	1, 15	5.80	0.0293	2, 30	0.06	0.9410	0.2123	0.0797	0.0511	0.0973	0.0146	0.9460	0.6133

Results of repeated-measures ANOVA (left), pairwise between-subject and paired within-subject Sidak-adjusted simple-main effects post-hoc tests (middle) for the experiments shown in Figure 2 conducted in PV-Cre mice transfected with hM3q and their mCherry-transfected controls. All repeated-measures ANOVAs are two-way ANOVAs involving one within-subject parameter (CNO or drug) and one between-subject parameter (subgroup); N-numbers are 9 mCh, 8 Gq. *Reasons for varying N-numbers compared to other experiments in the PV-Gq cohort:* One mCh-control and one Gq mouse were excluded from the Ro-challenge because they did not react to Ro (< 5% prematures under Ro alone), data from one further Gq mouse in this challenge was lost due to a technical error. The figure that displays the statistically tested data is shown in the figure panel indicated in the “Figure” column. The degrees of freedom (df1, df2) are indicated for the distinct effects of group, dose, and interaction. Post-hoc tests that were not indicated to be run because of a lack of significant between-subject, within-subject or interaction effect in the overall RM-ANOVA are omitted (grey cells). *P*-values < 0.01 are highlighted in dark green, *P*-values < 0.05 in light green, *P*-values < 0.1 in light grey-green.

Supplementary Table 5. CNO-induced 5-CSRTT behaviour in the PV-hM4Di cohort.

CNO				RM ANOVA							Sidak post-hoc tests: p-value			
Protocol, CNO dose		Behavioural parameter	Figure		dose		group		interaction		Gq vs Ctrl		Vehicle vs CNO	
				df1,df2	F	p	F	p	F	p	Vehicle	CNO	Gi	Ctrl
7s ITI		%premature	4d	1, 20	2.09	0.1633	1.81	0.1940	0.07	0.7875				
10 mg/kg		accuracy	4d	1, 20	0.35	0.5583	4.84	0.0398	3.21	0.0884	0.4248	0.0071	0.0920	0.4277
Group	N	%omissions	4d	1, 20	0.00	0.9469	0.27	0.6101	0.11	0.7484				
Ctrl	10	#correct	S11	1, 20	4.32	0.0508	1.24	0.2786	0.00	0.9527				
ACC-Gi	12	#incorrect	4d	1, 20	0.31	0.5858	1.93	0.1803	3.22	0.0881				
		%perseverative	-	1, 20	2.65	0.1193	0.03	0.8658	0.08	0.7797				
		reward latency	4d	1, 20	0.83	0.3721	2.07	0.1653	0.15	0.7042				
		response latency	4d	1, 20	2.35	0.1407	4.36	0.0498	1.29	0.2694	0.0282	0.1794		
9s ITI		%premature	4e	1, 18	3.99	0.0603	0.24	0.6334	0.19	0.6697				
10 mg/kg		accuracy	4e	1, 18	1.22	0.2823	0.34	0.5646	0.02	0.9017				
Group	N	%omissions	4e	1, 18	2.86	0.1073	0.10	0.7571	1.10	0.3067				
Ctrl	9	#correct	S11	1, 18	0.00	0.9519	1.19	0.2897	1.29	0.2696				
ACC-Gi	12	#incorrect	4e	1, 18	1.06	0.3165	0.00	0.9724	1.06	0.3165				
		%perseverative	-	1, 18	0.16	0.6912	0.05	0.8200	0.98	0.3339				
		reward latency	4e	1, 18	6.88	0.0167	1.20	0.2879	5.45	0.0307	0.6736	0.1091	0.0012	0.8506
		response latency	4e	1, 18	2.71	0.1161	1.48	0.2390	0.65	0.4298				
Baseline		%premature	4h	1, 20	1.39	0.2528	2.15	0.1583	3.13	0.0920				
10 mg/kg		accuracy	4h	1, 20	0.10	0.7588	2.27	0.1472	0.41	0.5296				
Group	N	%omissions	4h	1, 20	0.26	0.6188	0.46	0.5045	0.64	0.4346				
Ctrl	10	#correct	S11	1, 20	1.05	0.3178	1.57	0.2241	0.01	0.9267				
ACC-Gi	12	#incorrect	4h	1, 20	0.04	0.8414	2.16	0.1576	0.59	0.4501				
		%perseverative	-	1, 20	0.20	0.6617	0.76	0.3926	0.02	0.8843				
		reward latency	4h	1, 20	6.10	0.0226	0.13	0.7193	1.62	0.2173			0.3854	0.0197
		response latency	4h	1, 20	1.30	0.2684	3.71	0.0685	0.09	0.7670				
0.8 s SD		%premature	4f	1, 20	1.25	0.2777	0.34	0.5687	0.92	0.3484				
10 mg/kg		accuracy	4f	1, 20	0.94	0.3437	0.06	0.8124	0.61	0.4439				
Group	N	%omissions	4f	1, 20	0.06	0.8137	1.38	0.2538	1.62	0.2179				
Ctrl	10	#correct	S11	1, 20	1.00	0.3281	1.61	0.2189	0.04	0.8372				
ACC-Gi	12	#incorrect	4f	1, 20	0.79	0.3843	0.13	0.7233	1.31	0.2663				
		%perseverative	-	1, 20	0.00	0.9541	0.31	0.5809	0.45	0.5098				
		reward latency	4f	1, 20	0.35	0.5585	1.92	0.1806	1.06	0.3161				
		response latency	4f	1, 20	0.21	0.6496	2.30	0.1453	0.01	0.9346				
7s ITI & 0.8s SD		%premature	4g	1, 19	0.19	0.6712	0.02	0.9025	0.40	0.5355				
10 mg/kg		accuracy	4g	1, 19	7.18	0.0148	1.65	0.2140	0.22	0.6416			0.0264	0.1607
Group	N	%omissions	4g	1, 19	0.64	0.4323	2.06	0.1672	0.73	0.4036				
Ctrl	9	#correct	S11	1, 19	7.23	0.0146	3.80	0.0662	0.07	0.8012	0.2442	0.0372	0.0366	0.1241
ACC-Gi	12	#incorrect	4g	1, 19	0.93	0.3479	0.78	0.3871	0.01	0.9274				
		%perseverative	-	1, 19	1.82	0.1930	1.30	0.2685	1.16	0.2946				
		reward latency	4g	1, 19	1.99	0.1748	3.97	0.0608	1.42	0.2488				
		response latency	4g	1, 19	0.14	0.7131	0.02	0.8931	0.23	0.6396				

CLZ				RM ANOVA							Sidak post-hoc tests: p-value			
Protocol, CLZ dose		Behavioural parameter	Figure		dose		group		interaction		Gq vs Ctrl		Vehicle vs CNO	
				df1,df2	F	p	F	p	F	p	Vehicle	CNO	Gi	Ctrl
7s ITI		%premature	S11	1, 20	0.12	0.7380	0.56	0.4614	0.81	0.3802				
0.3 mg/kg		accuracy	S11	1, 20	0.76	0.3925	2.32	0.1432	0.40	0.5343				
Group	N	%omissions	S11	1, 20	0.53	0.4747	0.21	0.6549	0.14	0.7162				
Ctrl	10	#correct	S11	1, 20	0.01	0.9427	0.00	0.9709	0.43	0.5202				
ACC-Gi	12	#incorrect	S11	1, 20	1.23	0.2801	1.75	0.2003	0.32	0.5799				
		%perseverative	-	1, 20	0.85	0.3668	2.66	0.1187	1.36	0.2577				
		reward latency	S11	1, 20	5.57	0.0286	0.00	0.9570	1.15	0.2958			0.0192	0.3942
		response latency	S11	1, 20	2.06	0.1666	0.13	0.7235	0.64	0.4318				
Baseline		%premature	S11	1, 20	0.66	0.4276	0.77	0.3894	0.02	0.8864				
0.3 mg/kg		accuracy	S11	1, 20	0.22	0.6420	1.73	0.2027	0.01	0.9282				
Group	N	%omissions	S11	1, 20	0.78	0.3882	0.01	0.9104	0.50	0.4869				
Ctrl	10	#correct	S11	1, 20	2.14	0.1587	0.68	0.4200	0.73	0.4022				
ACC-Gi	12	#incorrect	S11	1, 20	0.01	0.9161	0.69	0.4162	0.01	0.9161				
		%perseverative	-	1, 20	5.52	0.0292	0.24	0.6276	0.15	0.7013			0.0558	0.6276
		reward latency	S11	1, 20	7.33	0.0136	0.07	0.7881	0.09	0.7649			0.0372	0.1194
		response latency	S11	1, 20	0.09	0.7624	0.33	0.5747	0.31	0.5824				
0.8 s SD		%premature	S11	1, 19	0.04	0.8375	0.96	0.3386	3.23	0.0881	0.7569	0.1487	0.1624	0.2857
0.3 mg/kg		accuracy	S11	1, 19	0.76	0.3943	1.06	0.3170	0.01	0.9299				
Group	N	%omissions	S11	1, 19	0.46	0.5063	0.05	0.8223	0.02	0.8998				
Ctrl	10	#correct	S11	1, 19	0.88	0.3587	0.11	0.7462	0.04	0.8357				
ACC-Gi	11	#incorrect	S11	1, 19	0.02	0.8821	1.84	0.1911	0.00	0.9556				
		%perseverative	-	1, 19	6.77	0.0175	0.46	0.5051	0.85	0.3679			0.2384	0.0249
		reward latency	S11	1, 19	1.26	0.2759	0.41	0.5277	0.46	0.5069				
		response latency	S11	1, 19	0.80	0.3832	0.03	0.8683	0.00	0.9960				
7s ITI & 0.8s SD		%premature	S11	1, 19	0.27	0.6080	1.04	0.3208	0.03	0.8663				
0.3 mg/kg		accuracy	S11	1, 19	1.03	0.3218	2.68	0.1182	0.37	0.5514				
Group	N	%omissions	S11	1, 19	0.81	0.3808	0.45	0.5106	2.03	0.1709				
Ctrl	9	#correct	S11	1, 19	7.13	0.0151	0.10	0.7551	5.47	0.0305	0.2324	0.6646	0.8023	0.0037
ACC-Gi	12	#incorrect	S11	1, 19	0.05	0.8329	1.20	0.2865	0.02	0.8852				
		%perseverative	-	1, 19	0.01	0.9393	3.39	0.0814	3.00	0.0996	0.0379	0.5295	0.2217	0.2464
		reward latency	S11	1, 19	0.00	0.9520	0.03	0.8549	0.20	0.6613				
		response latency	S11	1, 19	2.87	0.1063	0.97	0.3367	0.81	0.3784				

Results of repeated-measures ANOVA (left), pairwise between-subject and paired within-subject Sidak-adjusted simple-main effects post-hoc tests (middle) for the experiments shown in Fig. 4 and Supplementary Fig. 11 conducted in PV-Cre mice transfected with hM4Di and their mCherry-transfected controls. *Reasons for varying N-numbers:* In the 0.8s-SD challenge (CLZ), one hM4Di-mouse did not perform on one of the experimental days (>90% omissions) and was therefore excluded. One mCh-mouse had to be killed prematurely due to ill health (unrelated to procedures) and therefore did not participate in the two combined 7s-ITI/0.8s-SD challenges (CNO, CLZ) nor in the 9s-ITI challenge (CNO). All repeated-measures ANOVAs are two-way ANOVAs involving one within-subject parameter (CNO or CLZ) and one between-subject parameter (subgroup, identified in the small tables on the left with contributing N-numbers). The analysed challenge protocol and applied drug are identified at the top-left of each section. The figure that displays the statistically tested data is shown in the figure panel indicated in the "Fig." column. For all challenges, the degrees of freedom (df1, df2) are identical for all three factors of the ANOVA and hence only stated once. Post-hoc tests that were not indicated to be run because of a lack of significant between-subject, within-subject or interaction effect in the overall RM-ANOVA are omitted (grey cells). *P*-values < 0.01 are highlighted in dark green, *P*-values < 0.05 in light green, *P*-values < 0.1 in light grey-green.

Supplementary Table 6. Statistical assessment of CNO-induced 5-CSRTT behaviour in the Sst-Gq cohort.

Chemogenetic experiments in SST-Gq cohort

Protocol, CNO dose		Behavioural parameter	Figure	RM ANOVA							Sidak post-hoc tests: p-value			
					dose		group		interaction		Gq vs Ctrl		Vehicle vs CNO	
				df1,df2	F	p	F	p	F	p	Vehicle	CNO	Gq	Ctrl
**Baseline**		%premature	**S13**	1, 23	0.07	0.7999	0.22	0.6400	0.00	0.9887				
2.1 mg/kg		accuracy	**S13**	1, 23	0.07	0.7983	0.33	0.5735	0.87	0.3599				
**Group**	**N**	%omissions	**S13**	1, 23	2.60	0.1206	1.42	0.2457	0.91	0.3507				
Ctrl	15	#correct	**S13**	1, 23	1.56	0.2248	1.60	0.2181	0.91	0.3491				
Gq	10	#incorrect	**S13**	1, 23	0.10	0.7527	0.19	0.6684	0.80	0.3812				
		%perseverative	-	1, 23	0.98	0.3323	2.36	0.1381	0.22	0.6436				
		reward latency	**S13**	1, 23	2.62	0.1196	0.15	0.7025	2.02	0.1689				
		response latency	**S13**	1, 23	0.01	0.9144	1.22	0.2803	1.23	0.2784				
**7s ITI**		%premature	**5e**	1, 22	1.92	0.1795	0.00	0.9468	0.38	0.5454				
2.1 mg/kg		accuracy	**5e**	1, 22	4.29	0.0503	0.01	0.9095	0.01	0.9095			0.2291	0.0881
**Group**	**N**	%omissions	**5e**	1, 22	0.21	0.6480	0.53	0.4742	0.29	0.5954				
Ctrl	15	#correct	**S13**	1, 22	2.01	0.1703	2.14	0.1579	0.02	0.8854				
Gq	9	#incorrect	**5e**	1, 22	5.10	0.0342	0.18	0.6763	0.11	0.7399			0.2372	0.0457
		%perseverative	-	1, 22	0.10	0.7584	0.00	0.9980	0.70	0.4112				
		reward latency	**5e**	1, 22	0.99	0.3302	0.06	0.8048	0.51	0.4834				
		response latency	**5e**	1, 22	2.44	0.1326	0.18	0.6782	0.19	0.6658				
**7s ITI**		%premature	**5f**	1, 21	1.29	0.2686	0.12	0.7322	0.68	0.4184				
2.1 mg/kg		accuracy	**5f**	1, 21	0.51	0.4836	0.10	0.7521	0.41	0.5299				
**Group**	**N**	%omissions	**5f**	1, 21	0.06	0.8072	0.73	0.4010	1.21	0.2836				
Ctrl	14	#correct	**S13**	1, 21	0.24	0.6301	0.13	0.7171	0.40	0.5328				
Gq	9	#incorrect	**5f**	1, 21	0.74	0.4006	0.17	0.6804	0.13	0.7262				
		%perseverative	-	1, 21	1.09	0.3080	0.83	0.3730	0.19	0.6648				
		reward latency	**5f**	1, 21	6.62	0.0182	0.92	0.3477	0.00	0.9692			0.1279	0.0426
		response latency	**5f**	1, 21	0.11	0.7380	0.25	0.6228	2.65	0.1185				
**9s ITI**		%premature	**5g**	1, 21	2.74	0.1129	0.03	0.8697	1.18	0.2903				
10 mg/kg		accuracy	**5g**	1, 21	8.35	0.0088	1.04	0.3196	7.13	0.0143	0.0225	0.4241	0.0024	0.8538
**Group**	**N**	%omissions	**5g**	1, 21	7.77	0.0111	0.01	0.9073	2.91	0.1029			0.0140	0.2839
Ctrl	15	#correct	**S13**	1, 21	0.02	0.8772	1.27	0.2728	0.08	0.7742				
Gq	8	#incorrect	**5g**	1, 21	8.97	0.0069	0.70	0.4122	6.36	0.0198	0.0266	0.4547	0.0026	0.6915
		%perseverative	-	1, 21	0.23	0.6386	0.21	0.6509	2.50	0.1290				
		reward latency	**5g**	1, 21	2.87	0.1063	0.09	0.7683	0.30	0.5874				
		response latency	**5g**	1, 21	14.05	0.0012	0.83	0.3718	0.89	0.3567			0.0970	0.0007
**0.8 s SD**		%premature	**5h**	1, 23	1.90	0.1809	0.40	0.5325	0.06	0.8156				
2.1 mg/kg		accuracy	**5h**	1, 23	0.58	0.4531	1.98	0.1728	0.15	0.6995				
**Group**	**N**	%omissions	**5h**	1, 23	0.84	0.3684	0.31	0.5822	0.07	0.7968				
Ctrl	15	#correct	**S13**	1, 23	0.02	0.8903	1.23	0.2798	0.20	0.6614				
Gq	10	#incorrect	**5h**	1, 23	1.23	0.2787	0.74	0.3979	0.21	0.6489				
		%perseverative	-	1, 23	6.35	0.0192	0.34	0.5678	0.50	0.4865			0.0486	0.1655
		reward latency	**5h**	1, 23	6.79	0.0158	2.61	0.1198	5.89	0.0234	0.5694	0.0444	0.0035	0.8896
		response latency	**5h**	1, 23	0.00	0.9476	0.06	0.8151	3.66	0.0682	0.3808	0.5793	0.2454	0.1312
**0.8 s SD**		%premature	**5i**	1, 21	0.92	0.3474	1.12	0.3017	0.95	0.3420				
10 mg/kg		accuracy	**5i**	1, 21	0.30	0.5904	0.56	0.4625	0.22	0.6464				
**Group**	**N**	%omissions	**5i**	1, 21	0.24	0.6290	0.52	0.4790	0.51	0.4830				
Ctrl	15	#correct	**S13**	1, 21	0.12	0.7305	0.07	0.7999	1.64	0.2146				
Gq	8	#incorrect	**5i**	1, 21	0.83	0.3724	0.54	0.4722	0.08	0.7842				
		%perseverative	-	1, 21	1.56	0.2255	0.26	0.6155	0.78	0.3876				
		reward latency	**5i**	1, 21	13.61	0.0020	0.73	0.4059	5.26	0.0357	0.6557	0.0547	0.0021	0.2443
		response latency	**5i**	1, 21	0.12	0.7335	1.69	0.2088	0.46	0.5047				

Results of repeated-measures ANOVA (left), pairwise between-subject and paired within-subject Sidak-adjusted simple-main effects post-hoc tests (middle) for the experiments shown in Fig. 5 and Supplementary Fig. 13 conducted in SST-Cre mice transfected with hM3q and their mCherry-transfected controls. All repeated-measures ANOVAs are two-way ANOVAs involving one within-subject parameter (CNO) and one between-subject parameter (subgroup, identified in the small tables on the left with contributing N-numbers). *Reasons for varying N-numbers:* One Gq mouse participated only in the first three experiments (first 7s ITI, 0.8s SD,

baseline – each with 2.1 mg/kg CNO) due to unrelated health issues. A further Gq mouse did not participate in the first (7s ITI) challenge because it was delayed in training. One mCh-Control was excluded from the second 7s-ITI challenge with 2.1 mg/kg CNO, due to an error in the operant box which led to lack of participation on one of the test days (78% omissions); one Gq-mouse did not run in the 9s-ITI challenge with 10 mg/kg CNO, another one was excluded because it did not participate in the task (sedated appearance); two Gq-mice did not contribute data in the 0.8s-SD challenge with 10 mg/kg due to technical errors with the operant box. The analysed challenge protocol and applied drug are identified at the top-left of each section. The figure that displays the statistically tested data is shown in the figure panel indicated in the "Fig." column. For the parametric challenges, the degrees of freedom (df1, df2) are identical for all three factors of the ANOVA and hence only stated once. Post-hoc tests that were not indicated to be run because of a lack of significant between-subject, within-subject or interaction effect in the overall RM-ANOVA are omitted (grey cells). *P*-values < 0.01 are highlighted in dark green, *P*-values < 0.05 in light green, *P*-values < 0.1 in light grey-green.

Supplementary Table 7. Statistical assessment of CNO-induced 5-CSRTT behaviour in the Sst-Gq cohort, analysis across multiple challenges.

RM-ANOVA: combined analysis of 2nd and 3rd 7s-ITI challenges, 2.1 vs 10mg/kg CNO; Gq-mice only

**SST-Gq: n = 8**		**dose**		**drug**		**dose*drug**	
	**df1,df2**	**F**	**p**	**F**	**p**	**F**	**p**
%premature	1, 7	4.09	0.0827	2.51	0.1570	1.74	0.2287
accuracy	1, 7	2.98	0.1278	6.13	0.0424	8.28	0.0237
%omissions	1, 7	2.84	0.1355	1.89	0.2120	8.18	0.0243
#incorrect	1, 7	0.55	0.4818	6.79	0.0351	6.65	0.0365

Results of two-way RM-ANOVAs within the subgroups of Gq-transfected Sst-Cre mice involving data from two separate experiments, representing distinct dose-levels of CNO, for the experiments shown in Figure 5 and Supplementary Fig. 13 and identified at the top the table. The ANOVAs involve two within-subject parameters [drug (CNO vs. vehicle), and dose (0.7 vs. 2.1 mg/kg CNO)]. Reasons for varying *N*-numbers are as stated in the legend of Supplementary Table 6. The degrees of freedom (df1, df2) are identical for all factors of the ANOVA and hence only stated once. *P*-values < 0.01 are highlighted in dark green, *P*-values < 0.05 in light green, *P*-values < 0.1 in light grey-green.

Supplementary References

1 Kapanaiah SKT, van der Veen B, Strahnen D, Akam T, Kätzel D. A low-cost open-source 5-choice operant box system optimized for electrophysiology and optophysiology in mice. *Sci Rep* 2021; **11**: 22279.

2 Akam T, Lustig A, Rowland JM, Kapanaiah SK, Esteve-Agraz J, Panniello M *et al.* Open-source, Python-based, hardware and software for controlling behavioural neuroscience experiments. *eLife* 2022; **11**: e67846.

3 Grimm CM, Aksamaz S, Schulz S, Teutsch J, Sicinski P, Liss B *et al.* Schizophrenia-related cognitive dysfunction in the Cyclin-D2 knockout mouse model of ventral hippocampal hyperactivity. *Transl Psychiatry* 2018; **8**: 212.

4 Jendryka M, Palchaudhuri M, Ursu D, Veen B van der, Liss B, Kätzel D *et al.* Pharmacokinetic and pharmacodynamic actions of clozapine-N-oxide, clozapine, and compound 21 in DREADD-based chemogenetics in mice. *Sci Rep* 2019; **9**: 4522.

5 Bari A, Dalley JW, Robbins TW. The application of the 5-choice serial reaction time task for the assessment of visual attentional processes and impulse control in rats. *Nat Protoc* 2008; **3**: 759–767.

6 Schindelin J, Arganda-Carreras I, Frise E, Kaynig V, Longair M, Pietzsch T *et al.* Fiji - an Open Source platform for biological image analysis. *Nat Methods* 2012; **9**. doi:10.1038/nmeth.2019.

7 Strahnen D, Kapanaiah SKT, Bygrave AM, Kätzel D. Lack of redundancy between electrophysiological measures of long-range neuronal communication. *BMC Biol* 2021; **19**: 24.

8 Strahnen D, Kapanaiah SKT, Bygrave AM, Liss B, Bannerman DM, Akam T *et al.* Highly task-specific and distributed neural connectivity in working memory revealed by single-trial decoding in mice and humans. *bioRxiv* 2021; : 2021.04.20.440621.

9 Bokil H, Andrews P, Kulkarni JE, Mehta S, Mitra PP. Chronux: A platform for analyzing neural signals. *J Neurosci Methods* 2010; **192**: 146–151.

10 Vinck M, Oostenveld R, van Wingerden M, Battaglia F, Pennartz CMA. An improved index of phase-synchronization for electrophysiological data in the presence of volume-conduction, noise and sample-size bias. *NeuroImage* 2011; **55**: 1548–1565.

11 Oostenveld R, Fries P, Maris E, Schoffelen J-M. FieldTrip: Open Source Software for Advanced Analysis of MEG, EEG, and Invasive Electrophysiological Data. *Comput Intell Neurosci* 2010; **2011**. doi:10.1155/2011/156869.

12 Franklin K, Paxinos G. *The mouse brain in stereotaxic coordinates*. 3rd ed. Academic Press, 2007.
